# Prevalence and spectrum of variants in homologous recombination repair genes in ovarian cancer in Latin America: a systematic review and meta-analysis

**DOI:** 10.1016/j.xagr.2026.100693

**Published:** 2026-09-12

**Authors:** Elizabeth Vargas-Castellanos, Natalia Santiago-Tovar, Luisa Murcia-Soriano, Ariana Patino-Herrera, Rafael Parra-Medina, Julian C. Riano-Moreno

**Affiliations:** 1 Hospital Universitario Mayor—Méderi (Vargas-Castellanos and Murcia-Sonaiano), Universidad del Rosario. Bogotá D.C., Colombia; 2 Faculty of Medicine. Universidad Cooperativa de Colombia (Santiago-Tovar and Riano-Moreno), Villavicencio, Colombia; 3 Faculty of Medicine. Universidad Nacional de Colombia (Patino-Herrera), Bogotá D.C., Colombia; 4 Department of Pathology and Molecular Oncology (Parra-Medina and Riano-Moreno), Instituto Nacional de Cancerología, Bogotá D.C., Colombia; 5 Department of Pathology (Parra-Medina), Fundación Universitaria de Ciencias de la Salud - FUCS, Bogota D.C., Colombia

**Keywords:** *BRCA1*, *BRCA2*, homologous recombination repair, HRR gene, Latin America, non-BRCA, ovarian cancer

## Abstract

**BACKGROUND:**

Ovarian cancer is a major cause of gynecologic cancer mortality worldwide, with disproportionately poor outcomes in Latin America (LATAM). Variants in homologous recombination repair genes, especially *BRCA1/2*, are predictive biomarkers for platinum-based chemotherapy and PARP inhibitors. However, data on the prevalence and spectrum of homologous recombination repair variants in LATAM populations remain limited.

**OBJECTIVE:**

To synthesize the prevalence and spectrum of pathogenic and likely pathogenic variants in homologous recombination repair pathway genes among LATAM ovarian cancer patients and highlight clinical and research implications.

**STUDY DESIGN:**

The review protocol was developed in accordance with the Preferred Reporting Items for Systematic Reviews and Meta-Analyses (PRISMA) and was registered in the international prospective register of systematic reviews (PROSPERO; registration number CRD420251019706). We systematically searched MEDLINE, EMBASE, Scopus, Web of Science, LILACS, Cochrane, and Epistemonikos through April 2025. Eligible studies included LATAM ovarian cancer patients tested with multigene panels covering homologous recombination repair genes beyond *BRCA1/2*. Data extraction followed 2025 ACMG/AMP guidelines, and study quality was assessed with the Joanna Briggs Institute checklist. Pooled prevalence was estimated using random- and common-effects meta-analysis.

**RESULTS:**

Eight studies published between 2017 and 2023, including 442 patients with ovarian cancer from Brazil, Argentina, Mexico, Chile, and Colombia, were included in the systematic review. Following variant curation and reclassification, 7 studies comprising 433 patients and 96 pathogenic/likely pathogenic homologous recombination repair variant carriers were retained for prevalence analysis, yielding a crude prevalence of 22.2%. Pathogenic variants predominated (91.7%), with *BRCA1* (55.2%) and *BRCA2* (40.6%) accounting for ∼96% of carriers. Non-*BRCA* homologous recombination repair variants (*CHEK2, BARD1, and RAD51D*) accounted for 4.2%. The pooled prevalence of germline pathogenic/likely pathogenic homologous recombination repair variants was 17% (95% CI, 9%–26%; I²=56.2%), increasing to 20% (95% CI, 16%–23%; I²=0%) after sensitivity analysis excluding 2 micro-cohorts. Five recurrent variants were identified across multiple cohorts and countries. No study evaluated somatic homologous recombination repair variants or homologous recombination deficiency assays

**CONCLUSION:**

This first systematic synthesis of homologous recombination repair variants in LATAM ovarian cancer reveals a predominance of *BRCA1/2* but also underscores the relevance of non-*BRCA* homologous recombination repair genes. Lack of somatic testing, methodological heterogeneity, and limited regional coverage highlight the need for standardized approaches, multicenter collaboration, and equitable access to genomic diagnostics in the region.

**Implications for Practice:**

This systematic review highlights both opportunities and barriers for implementing homologous recombination repair testing in ovarian cancer across Latin America. While *BRCA1/2* remains the predominant genes, non-*BRCA* variants are also clinically relevant. Importantly, we identified substantial inequities in the distribution of genomic testing capacity, with most data concentrated in Brazil and Argentina, leaving many countries unrepresented. The absence of somatic homologous recombination repair/homologous recombination deficiency testing and heterogeneous sequencing approaches further limit regional comparability and access to precision therapies. Addressing these disparities through standardized testing strategies and collaborative networks is critical to ensure equitable access to genetic counseling and targeted treatments.

**AJOG Global Reports at a Glance**

**Why was this study conducted**

To determine the prevalence and spectrum of homologous recombination repair gene variants in ovarian cancer across Latin America through a systematic review and meta-analysis. To identify current evidence gaps relevant to precision oncology implementation in the region.

**Key findings**

Eight studies including 442 patients from 5 Latin American countries were analyzed. The pooled prevalence of pathogenic/likely pathogenic homologous recombination repair variants was 17%, increasing to 20% after sensitivity analysis; *BRCA1* and *BRCA2* accounted for approximately 96% of all variants, whereas non-*BRCA* homologous recombination repair variants were uncommon. No study evaluated somatic homologous recombination repair testing or homologous recombination deficiency.

**What does this add to what is known?**

This is the first systematic review and meta-analysis summarizing homologous recombination repair gene variants in ovarian cancer across Latin America. The findings support expanding standardized homologous recombination repair testing, including non-*BRCA* genes, and highlight the need for equitable access to genomic diagnostics and multicenter research in the region.

## Introduction

Ovarian cancer remains one of the deadliest malignancies in women, with approximately 207,000 deaths annually worldwide,^1^ primarily due to late-stage diagnosis; nearly 80% of patients present with disseminated disease and only ∼20% diagnosed at early stages.^2^^,^^3^ Consequently, the 5-year relative survival remains approximately 45%, dropping to <35% at 10 years.^2^, ^3^, ^4^ In Latin America (LATAM), the age-standardized incidence and mortality are 5.7 and 3.6 per 100,000, respectively, with Uruguay, Colombia, Argentina, and Venezuela reporting the highest national rates, though still intermediate compared with global figures.^1^

Given the poor outcomes associated with late detection, therapeutic advances in advanced OC have increasingly relied on molecular biomarkers to guide targeted therapies. In this context, the identification of alterations in homologous recombination repair (HRR) genes has become particularly relevant, as these variants predict sensitivity to platinum compounds and PARP inhibitors, offering treatment alternatives beyond conventional chemotherapy.^5^, ^6^, ^7^, ^8^, ^9^, ^10^

HRR safeguards genome integrity by repairing double-strand DNA breaks. Numerous genes participate in this pathway, including sensors of DNA damage (*γH2AX, ATM, ATR*), signal mediators (*BRCA1, BRCA2, PALB2*), and the effector RAD51, which promotes strand invasion and replication fork stabilization. Among them, *BRCA1* and *BRCA2* are the most extensively characterized, with well-established associations with breast, ovarian, pancreatic, and prostate cancers.^11^^,^^12^ Despite their central role, there is no consensus on which additional HRR genes should be systematically evaluated. Multigene panels vary widely in design—some limited to *BRCA1/2* as surrogates of homologous recombination deficiency (HRD), others extending to a broader set of HRR-related genes in either germline or somatic contexts.^13^, ^14^, ^15^, ^16^, ^17^ This variability complicates prevalence estimates and hinders cross-study comparisons, representing a critical methodological limitation.

Historically, clinical guidelines and therapeutic trials concentrated on *BRCA1/2*, given their established predictive value for platinum sensitivity and PARP inhibitor benefit.^5^^,^^6^^,^^9^^,^^10^^,^^18^^,^^19^ However, growing evidence indicates that other HRR genes—including *RAD51C, RAD51D, PALB2, CHEK2*, and *BARD1*—also contribute to hereditary ovarian cancer predisposition and may influence therapeutic response.^20^, ^21^, ^22^, ^23^, ^24^, ^25^, ^26^, ^27^ This recognition highlights the importance of evaluating the full spectrum of pathogenic (P) and likely pathogenic (LP) HRR variants rather than focusing exclusively on *BRCA1/2*.^18^^,^^28^^,^^29^

LATAM is characterized by extensive admixed ancestry involving Indigenous, European, and African lineages, shaped by migration and founder effects that may generate unique variant spectra.^30^, ^31^, ^32^ Despite this diversity, most regional research remains concentrated in a few countries, particularly Brazil,^33^ leaving major gaps in the systematic assessment of HRR variants across the continent. Addressing this evidence gap is essential for improving genetic counseling, informing prevention strategies, and expanding access to targeted therapies tailored to the genetic diversity of the region.^34^, ^35^, ^36^, ^37^

Therefore, this systematic review aims to synthesize the prevalence and spectrum of P and LP variants in HRR pathway genes among ovarian cancer patients in LATAM, highlighting their clinical relevance and the need for collaborative genomic research in the region.

## STUDY DESIGN

### Protocol and gene selection

The review protocol was developed in accordance with the Preferred Reporting Items for Systematic Reviews and Meta-Analyses (PRISMA)^38^ and was registered in the international prospective register of systematic reviews (PROSPERO; registration number CRD420251019706). Given the heterogeneity of HRR genes reported across commercially available companion diagnostic kits and international guidelines, we first reviewed the gene content of 8 commercial tests^39^, ^40^, ^41^, ^42^, ^43^, ^44^, ^45^, ^46^ (Supplemental Table 1) and 4 international guidelines recommending HRR/HRD testing^18^^,^^29^^,^^47^^,^^48^ (Supplemental Table 2). Genes were considered recurrent if they appeared in at least 5 of these sources (Supplemental Table 3). This process led to the identification of 15 HRR genes—*BRCA1, BRCA2, PALB2, BRIP1, RAD51C, RAD51D, BARD1, ATM, CHEK2, CHEK1, CDK12, NBN, FANCL, RAD51B*, and *RAD54L*—which were subsequently used to design the search strategy.

### Search strategy

The systematic search was conducted in MEDLINE, EMBASE, LILACS, The Cochrane Library, Scopus, Web of Science, and Epistemonikos (Supplemental Table 4), covering all records from database inception through April 2025. No restrictions regarding language or publication period were applied. A preliminary search was first performed in MEDLINE (Ovid) to refine the strategy, which was subsequently adapted to the other databases. In addition, reference lists of the included studies were screened to identify further eligible articles. The complete search strategies are presented in Supplemental Table 4.

### Study selection

The selection process was conducted in 2 stages. First, titles and abstracts were independently screened by 2 reviewers (EVC, LMS) against the predefined inclusion criteria. Second, potentially eligible studies were uploaded to Rayyan^49^ for full-text screening. Discrepancies were resolved by discussion or adjudication by a third reviewer (JCR).

Eligible studies were quantitative observational studies, including cohort, case-control, and analytical cross-sectional designs, that reported data on ovarian cancer patients from LATAM populations and assessed variants in HRR pathway genes using multigene panels. Studies enrolling patients with multiple cancer types, including hereditary breast and ovarian cancer (HBOC) cohorts, were eligible for inclusion if data for patients with ovarian cancer could be separately identified and extracted. No minimum proportion of ovarian cancer cases was required at the study level; however, studies in which ovarian cancer cases could not be distinguished from patients with other cancer types were excluded. Eligibility was assessed at the patient level whenever studies included mixed cancer populations. Only patients with a diagnosis of ovarian cancer contributed to the prevalence estimates and variant analyses. Patients diagnosed exclusively with other malignancies, including breast cancer, were not included in the analysis.

For HBOC cohorts, eligibility was determined by cancer diagnosis rather than by the presence of a hereditary syndrome, family history, or carrier status. Thus, only participants with ovarian cancer who met the predefined patient-level criteria were included, whereas participants with breast cancer or other malignancies were excluded from the analysis. When studies reported combined genetic results for breast and ovarian cancer without providing ovarian cancer-specific data, these results were not used to estimate HRR variant prevalence in ovarian cancer.

Studies in which genetic testing was restricted exclusively to *BRCA1/2* were excluded because the objective of this review was to characterize the prevalence and spectrum of HRR variants in the context of multigene testing. Eligible studies were required to evaluate *BRCA1/2* together with at least 1 additional HRR gene. This criterion was applied at the study level and did not exclude *BRCA1/2* variants from the analysis; therefore, *BRCA1/2* carriers identified within eligible multigene-testing cohorts were fully retained. The composition and size of the gene panels varied across studies, and the analysis was harmonized using the prespecified set of HRR genes described above. The study selection process is summarized in the PRISMA flow diagram (Figure 1).

**Figure 1:**
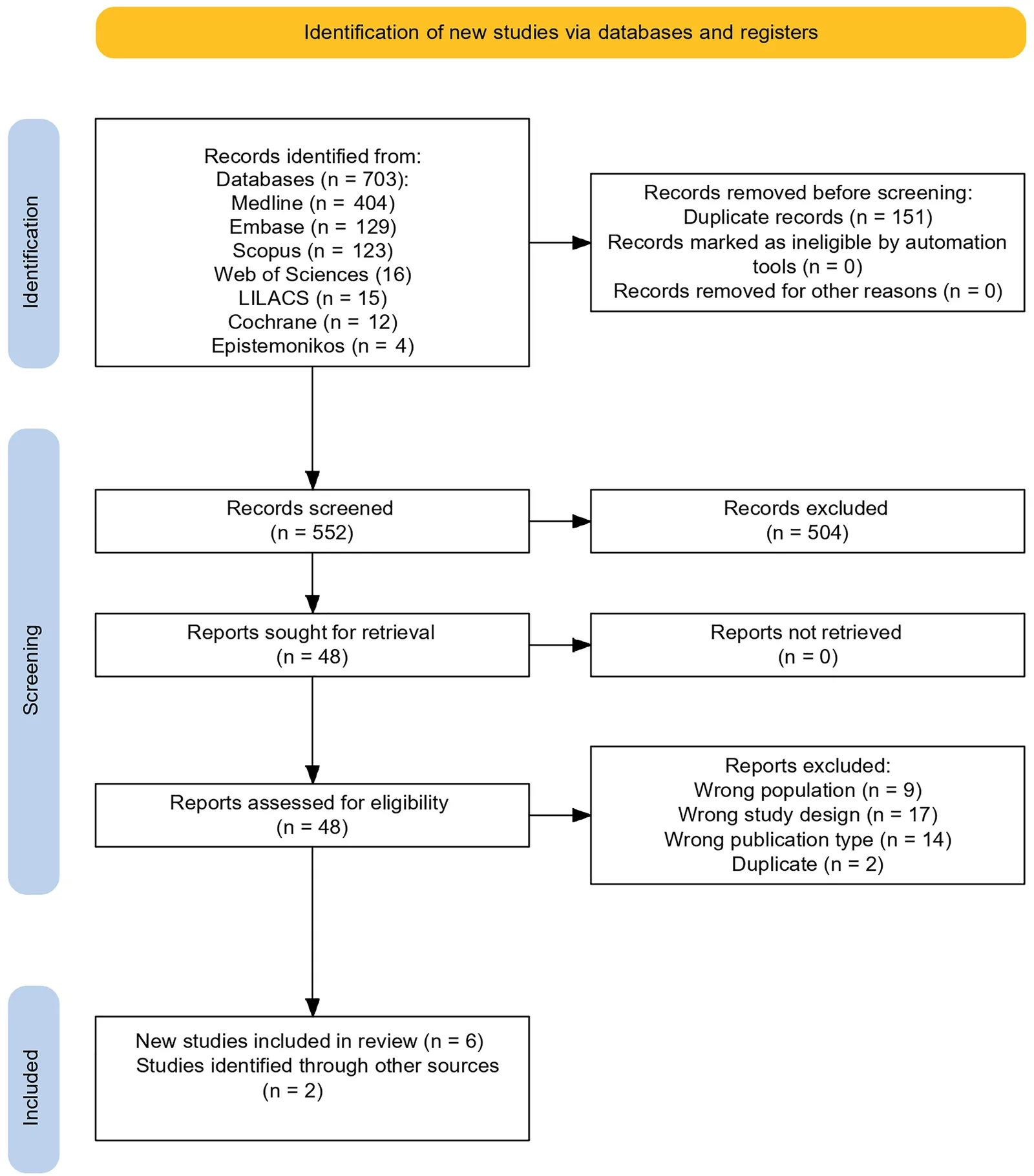
Flowchart of the systematic review process.^92^

Vargas-Castellanos. Prevalence and spectrum of variants in homologous recombination repair genes in ovarian cancer in Latin America: a systematic review and meta-analysis. Am J Obstet Gynecol 2026.

### Risk of bias assessment

The methodological quality and risk of bias of the included studies were independently assessed by 2 reviewers (APH, NS) using an adapted version of the Joanna Briggs Institute Critical Appraisal Checklist for Systematic Reviews and Research Syntheses.^50^ Discrepancies between reviewers were resolved by consensus or by consulting a third reviewer (EVC, JCR). The outcomes of this assessment are presented in Supplemental Figures 1 and 2.

### Data extraction

Data extraction was conducted independently by 2 reviewers (NS, APH) using a piloted Microsoft Excel template. Only data from patients diagnosed exclusively with ovarian cancer were included. Cases reporting patients with multiple primary tumors or a history of other cancers, were excluded to ensure that the analysis reflected solely the spectrum of variants associated with ovarian cancer. Extracted information included study characteristics such as authors, year, country, population size, ethnicity, age, and family history of cancer, as well as methodological features such as sequencing technique, platform, and the type and size of the gene panel used. Clinical data were collected regarding the number of participants, the number with ovarian cancer, and the number of ovarian cancer cases carrying HRR variants. Genetic data included reported cDNA and protein nomenclature, variant classifications, and whether the variants were of germline or somatic origin. All variants were curated according to the 2025 American College of Medical Genetics and Genomics/ Association for Molecular Pathology (ACMG/AMP) guidelines, and only those classified as P/LP were included in the final analysis.

### Data synthesis and statistical analysis

Descriptive statistics were used to summarize study characteristics and to quantify the frequencies and distribution of HRR variants. A random-effects meta-analysis was conducted to calculate pooled prevalence estimates, and study-specific outcomes with 95% confidence intervals were presented in forest plots. Statistical heterogeneity was assessed using Cochran’s Q test and quantified with the I² statistic, reported with its 95% confidence interval together with τ². Robustness was evaluated through influence diagnostics (Baujat plot), leave-one-out analysis, and re-estimation with a random-intercept logistic regression model, which accommodates sparse data and boundary proportions without continuity corrections. Potential sources of heterogeneity were explored in subgroup analyses by recruitment setting, country, breadth of the sequencing strategy, cohort size, and sequencing platform; because several subgroups contained cohorts of fewer than 5 patients, subgroups were summarized as crude prevalences with exact binomial confidence intervals and compared by likelihood-ratio test. Meta-regression was not performed, as fewer than 10 studies were available; all subgroup analyses are exploratory. Publication bias was examined descriptively through funnel plots; formal testing of funnel asymmetry is not recommended with fewer than 10 studies, and, in meta-analyses of proportions, asymmetry may arise artefactually from the dependence of the variance on the estimated proportion. All analyses were performed in R (version 4.5.1), using various packages for data visualization and the *meta* and *metaphor* packages for meta-analysis.

## Results

### Study selection

Following the predefined search strategy, a total of 703 records were retrieved across 7 databases (Medline, Embase, Scopus, Web of Science, LILACS, Cochrane, and Epistemonikos) (Supplemental Table 5). After removal of 151 duplicates, 552 unique records were screened by title and abstract, yielding 48 articles for full-text review. Of these, 42 were excluded—most commonly due to ineligible study design (n=17), publication type (n=14), or study population (n=9)—and 2 additional reports were duplicates. Ultimately, 6 studies were identified through the systematic database search, and 2 additional eligible studies were identified through other sources, resulting in a total of 8 independent studies included in the review (Figure 1). Although the search strategy was designed to capture both germline and somatic HRR studies, all included reports evaluated germline DNA, and none assessed somatic HRR variants in ovarian cancer patients from LATAM.

### Study-level characteristics

The 8 included studies, published between 2017 and 2023, enrolled 442 ovarian cancer patients from 5 countries: Brazil (n=4 studies, 86 patients), Argentina (n=1 study, 340 patients), Mexico (n=1 study, 9 patients), Chile (n=1 study, 1 patient), and Colombia (n=1 study, 6 patients) (Table 1). Brazil contributed half of the included studies, while Argentina accounted for the largest single cohort (77% of all patients) (Figure 2A).

**Table:** Characteristics of studies included in the systematic review on pathogenic and likely pathogenic variants in HRR pathway genes among ovarian cancer patients in LATAM.

Study (ref)	Country	Design/Recruitment setting	N ovarian cancer patients	Mean age (y)	Ancestry/Ethnicity reported	Panel/Platform	N° genes tested	N° carriers (genes reported)
Pérez-Ibave et al.^51^	Mexico	Cross-sectional/Hereditary clinic	9	36	Hispanic, LATAM/Caribbean, European, African, North American descent	Multi-Cancer Panel (Illumina)	84	2 (*BRCA1*)
De Carvalho et al.^52^	Brazil	Cross-sectional/Hereditary clinic	10	36	LATAM/Caribbean, European descent	*BRCA1/2* and multipanel (platform not reported)	Not specified	1 (*BRCA1*)
De Oliveira et al.^57^	Brazil	Cross-sectional/Hospital-based	64	NR	LATAM/Caribbean, European descent	Expanded panel (Illumina)	37–143	11 (*BRCA1*=7; *BRCA2*=2; *CHEK2*=1; *BARD1*=1)
Solano et al.^53^	Argentina	Cross-sectional/Hospital-based	340^a^	52.6	Asian, European, African, LATAM/Caribbean, North American descent	WES + MLPA (Illumina)	WES	77 (*BRCA1*=41; *BRCA2*=36)
Felicio et al.^54^	Brazil	Cross-sectional/Hospital-based	9	21	Brazilian	WES (Illumina)	WES	1 (*CHEK2*)
Adaniel et al.^56^	Chile	Cross-sectional/Hospital-based	1	50	Multiple ethnicities	NGS (platform not reported)	Non-*BRCA1/2* multipanel	1 (*RAD51D*)
Moreira et al.^55^	Brazil	Cross-sectional/Hospital-based	3	47	European, LATAM/Caribbean, North America	SANGER-seq (Applied Biosystems)	*TP53, CHEK2, BRCA1/2*	3 (*BRCA1*=1; *BRCA2*=1; *CHEK2*=1)
Cock-Rada et al.^58^	Colombia	Cross-sectional/Hospital-based	6	NR	LATAM/Caribbean	myRisk (Illumina)	25	1 (*BRCA1*)

*MLPA*, multiplex ligation-dependent probe amplification; *NGS*, next-generation sequencing; *NR*, not reported; *WES*, whole-exome sequencing.

a Information not reported in the cited article; this was reported in a previous publication from the same group.^93^ *Vargas-Castellanos. Prevalence and spectrum of variants in homologous recombination repair genes in ovarian cancer in Latin America: a systematic review and meta-analysis. Am J Obstet Gynecol 2026*.

**Figure 2:**
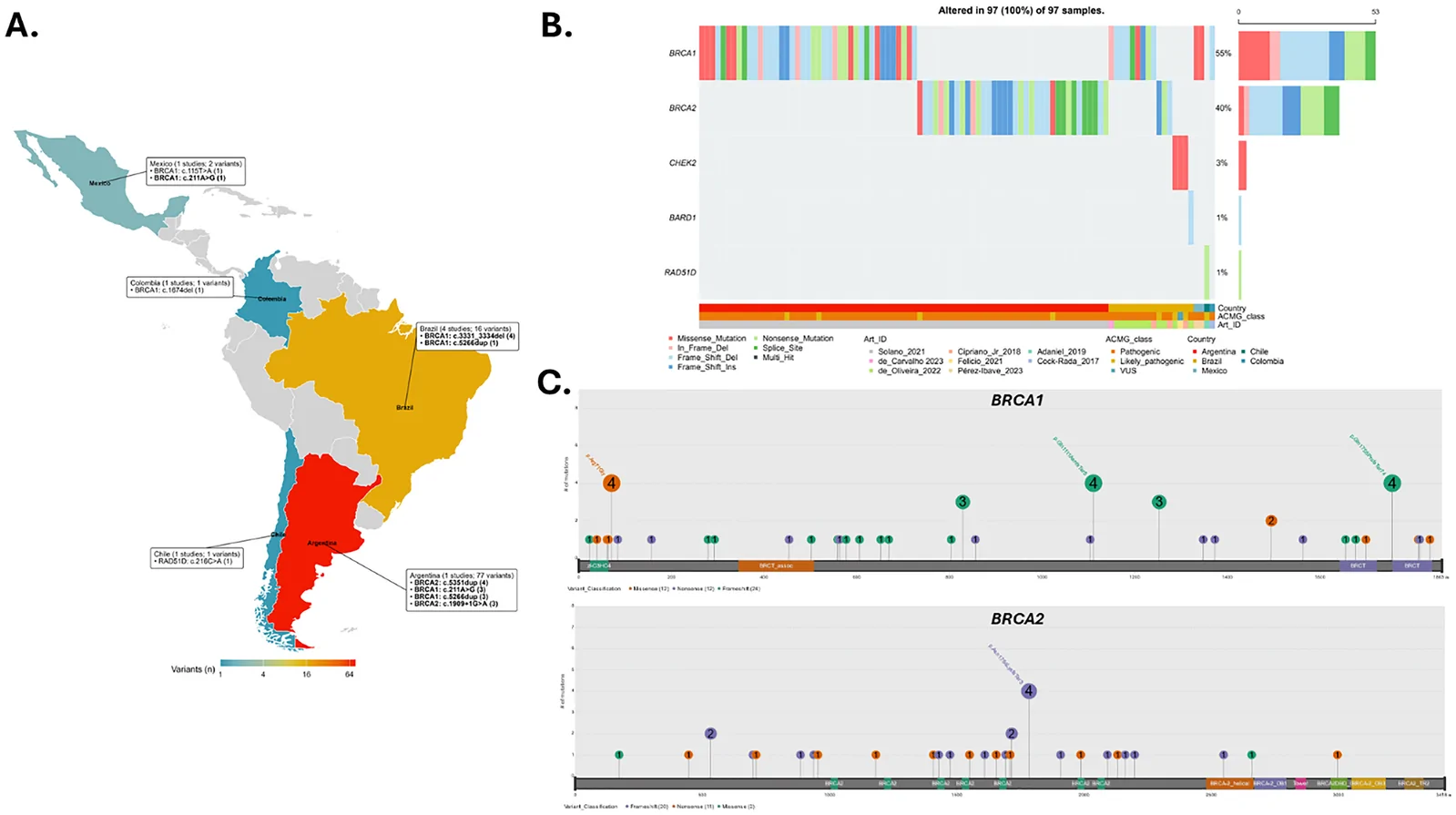
Distribution and spectrum of HRR gene variants in ovarian cancer patients from LATAM (A) Geographic distribution of included cohorts and reported HRR variants across LATAM countries. Variants in bold correspond to recurrent alterations and their distribution is indicated. (B) Oncoprint summarizing the spectrum of variants in HRR genes. (C) Lollipop plots of *BRCA1* and *BRCA2* variants showing their location within functional protein domains. Only recurrent variants are annotated. The recurrent *BRCA2* splice-site variant c.1909+1G>A is not shown, as splicing variants are not graphically represented in this format.

Vargas-Castellanos. Prevalence and spectrum of variants in homologous recombination repair genes in ovarian cancer in Latin America: a systematic review and meta-analysis. Am J Obstet Gynecol 2026.

Six studies were hospital-based, reflecting clinical oncology practice, whereas 2 were conducted in hereditary cancer clinics (Pérez-Ibave et al.^51^ de Carvalho et al.^52^). Most cohorts described admixed ancestry, predominantly LATAM/Caribbean and European descent, with additional contributions from African, Asian, North American, and Brazilian populations Supplemental Table 6.

Sequencing approaches were heterogeneous. Panels ranged from targeted *BRCA1/2* analysis plus selected non-*BRCA* HRR genes to expanded multigene panels covering up to 143 genes. Whole-exome sequencing (WES) was employed in 2 studies (Solano et al.^53^ Felicio et al.^54^). Other strategies included commercial multigene panels (eg, Invitae Multi-Cancer, myRisk), Sanger sequencing (Moreira et al.^55^) and unspecified next generation sequencing (NGS) (Adaniel et al.^56^).

Reporting familial history of cancer was inconsistent. Of the 8 studies, only 5 provided information on this variable. In Solano et al.,^53^ family history was noted but without details of specific cancers. In contrast, de Oliveira et al.,^57^ Moreira et al.,^55^ and Cock-Rada et al.^58^ reported specific familial cancer diagnoses. Breast cancer was the most frequently reported familial cancer, while ovarian cancer and other cancer types were reported less frequently (Supplemental Table 6). However, the available information was insufficient to consistently classify participants according to a HBOC pattern.

Data on age at diagnosis were available in six of the eight studies, corresponding to 86 carriers. Two studies did not provide age information. The reported ages reflected age at ovarian cancer diagnosis, with a mean of 53.4 years (median 54, range 21–77) (Supplemental Table 6). Notably, none of the included studies stratified results by ovarian cancer histological subtype, limiting assessment of variant prevalence across tumor subgroups.

### Variant-level findings

Across the 8 eligible studies, 97 HRR variant carriers were initially reported (Supplemental Table 7). All reported variants were manually curated. One variant initially classified as LP, the *CHEK2* missense variant c.538C>T (p.Arg180Cys) reported in the Brazilian cohort by Felicio et al.,^54^ was reclassified as a variant of uncertain significance (VUS) based on PM5_supporting and PP3 criteria. Because this was the only P/LP HRR variant reported in that cohort, the study no longer contributed any eligible P/LP variant to the prevalence analysis and was therefore excluded from the quantitative synthesis. The Felicio et al.^54^ cohort comprised 9 patients with ovarian cancer; consequently, the prevalence analysis included the remaining 7 studies, comprising 433 patients and 96 P/LP HRR variant carriers, corresponding to a crude prevalence of 22.2%. Among these carriers, P variants predominated (n=88; 91.7%), with a smaller proportion LP variant (n=8; 8.3%).

Among the 96 P/LP HRR variant carriers identified in the 7 studies retained for prevalence analysis (Figure 2B), *BRCA1* accounted for 53 carriers (55.2%) and *BRCA2* for 39 carriers (40.6%), together representing 95.8% of all HRR carriers. Non-*BRCA* HRR genes contributed only 4 carriers (4.2%): *CHEK2* (n=2, excluding the reclassified variant), *BARD1* (n=1), and *RAD51D* (n=1).

By molecular consequence, frameshift variants were most frequent (n=45; 46.9%), followed by nonsense (n=24; 25.0%), missense (n=16; 16.7%), splice-site variants (n=10; 10.4%), and a single large deletion/indel (n=1; 1.0%), corresponding to a *BRCA1* whole-gene deletion c.(?-232)(1383_?)del. Stratified by gene category, *BRCA1/2* variants were dominated by frameshift and nonsense alterations (72.8%), whereas the few non-*BRCA* HRR variants included 1 frameshift (*BARD1*), 2 missense (*CHEK2*), and 1 nonsense (*RAD51D*).

### Recurrent variants

For this review, recurrent variants were defined as those detected in more than 2 carriers across the cohort. Five such variants were identified, all involving *BRCA1* or *BRCA2* (Figure 2C). In *BRCA1*, c.211A>G (p.Arg71Gly) was found in 4 carriers from Mexico (Pérez-Ibave., et al.^51^) and Argentina (Solano et al.^53^); c.3331_3334del (p.Gln1111AsnfsTer5) in 4 carriers across 2 Brazilian studies (de Oliveira et al.,^57^ Moreira et al.^55^); and c.5266dup (p.Gln1756ProfsTer74) in 4 carriers from Brazil and Argentina.^53^^,^^57^ In *BRCA2*, c.5351dup (p.Asn1784LysfsTer3) occurred in 4 carriers from Argentina (Solano et al.^53^), and the splice donor variant c.1909+1G>A was detected in 4 carriers from the same cohort.

### Meta-analysis of P/LP variant prevalence

To account for between-study variability, we performed a random-effects meta-analysis of germline P/LP variants. The pooled prevalence was 17% (95% CI, 9%–26%), with a 95% prediction interval of 11-23% (Figure 3A). Between-study heterogeneity was moderate in magnitude but imprecisely estimated (I²=56.2%, 95% CI 0.0%–81.2%; τ²=0; Q=13.69, df=6, *P*=.033); the confidence interval for I² includes zero, indicating that with 7 cohorts this statistic cannot reliably distinguish moderate inconsistency from none. This estimate was slightly lower than the crude prevalence of 22.2%, reflecting the weighting applied across studies.

**Figure 3:**
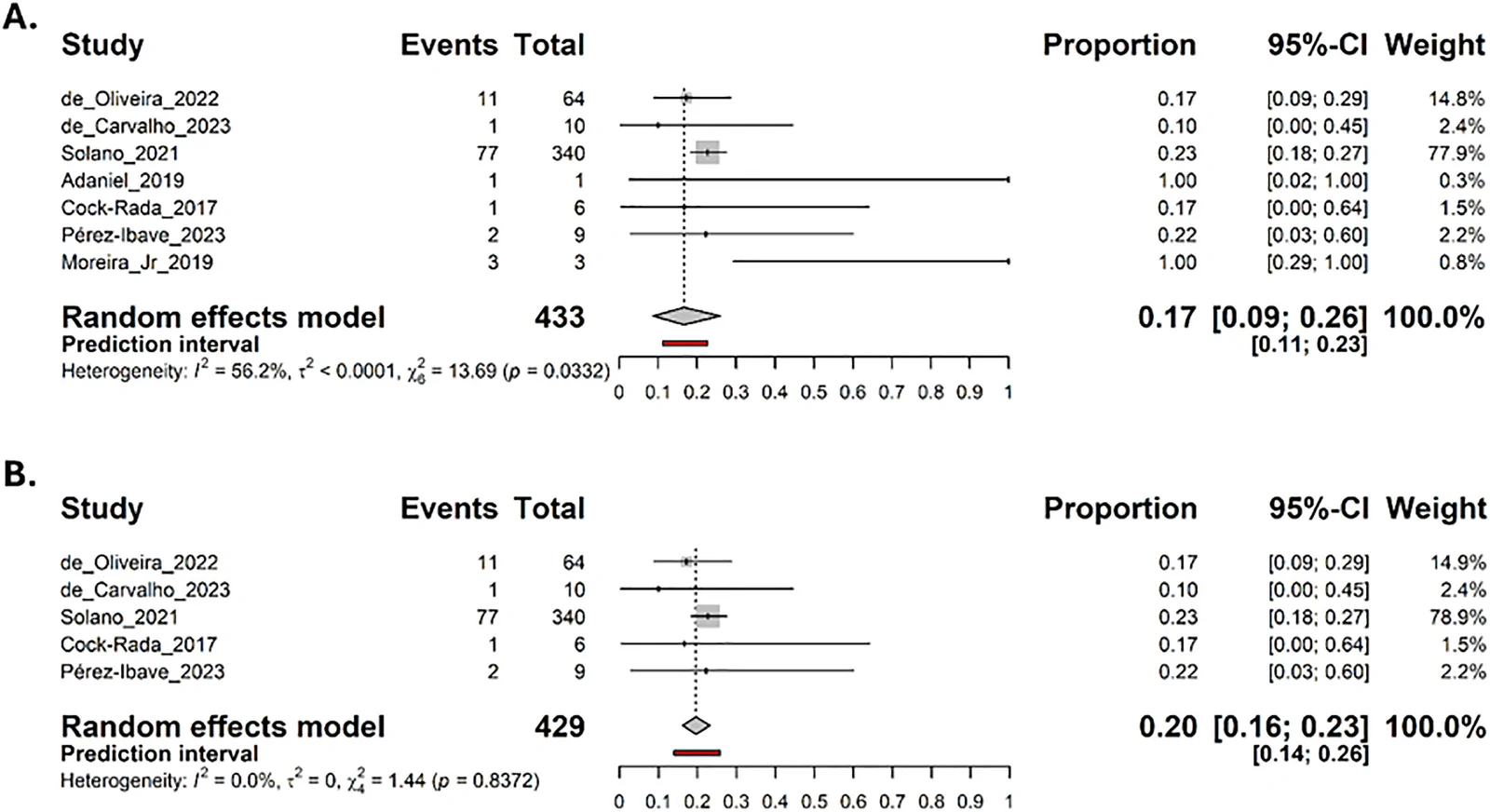
Forest plots of the prevalence of pathogenic/likely pathogenic germline variants in HRR genes among LATAM ovarian cancer cohorts (A) Study-specific prevalences and the pooled estimate from a random-effects meta-analysis, shown with 95% confidence intervals; the diamond marks the pooled prevalence and the red bar the 95% prediction interval. (B) Sensitivity analysis (“drop-2”) excluding the 2 cohorts of fewer than 5 patients (Adaniel et al.^56^ and Moreira et al.^55^), with 95% confidence intervals and the pooled estimate from the random-effects model.

Vargas-Castellanos. Prevalence and spectrum of variants in homologous recombination repair genes in ovarian cancer in Latin America: a systematic review and meta-analysis. Am J Obstet Gynecol 2026.

Sensitivity analyses excluded the 2 micro-cohorts contributing fewer than 5 patients, a criterion defined a priori and corroborated by influence diagnostics and leave-one-out analysis. After removing these 2 micro-studies (Adaniel et al.^56^ and Moreira et al.^55^ the pooled prevalence rose to 20% (95% CI, 16%–23%), with a prediction interval of 14-26%, and heterogeneity was eliminated (I²=0%) (Figure 3B). Re-estimation with a random-intercept logistic regression model, which handles sparse data and boundary proportions without transformation, yielded a pooled prevalence of 22.2% (95% CI, 17.7%–27.4%) with an estimated between-study variance of zero, indicating that the inconsistency observed under the double-arcsine model reflects the transformation of very small denominators rather than genuine variation in prevalence. Exploratory subgroup analyses identified no additional sources of heterogeneity: prevalence was comparable by recruitment setting (15.8% vs 22.5%; *P*=.48), country (19.5% vs 22.8%; *P*=.53), breadth of the sequencing strategy (21.8% vs 30.0%; *P*=.40), and cohort size (27.6% vs 21.8%; *P*=.48). Sequencing platform could not be assessed, as it was not reported in 2 of the 7 cohorts and only 1 cohort used a non-Illumina platform. With 1 to 5 studies per subgroup these comparisons had very limited power and are presented as exploratory (Supplemental Table 8). Gene specific prevalence was also assessed. To reduce small-study effects, the Moreira et al.^55^ cohort was excluded from this analysis. Only genes evaluated in at least 2 independent cohorts were considered, and common-effect models were applied. *BRCA1* showed a pooled prevalence of 10% (95% CI, 7%–13%) across 5 cohorts (n=429), while *BRCA2* had a prevalence of 9% (95% CI, 6%–12%) across 2 cohorts (n=404) (Figure 4A and B). Prediction intervals were not calculated for these gene-level analyses given the fixed-effect approach. Data for heterogeneity was absent for BRCA1 (I²=0%) but substantial for *BRCA2* (I²=75.4%); with only 2 contributing cohorts a between-study variance cannot be estimated, and the *BRCA2* estimate should therefore be regarded as descriptive. *CHEK2* and other non-*BRCA* HRR genes were insufficient for reliable pooling.

**Figure 4:**
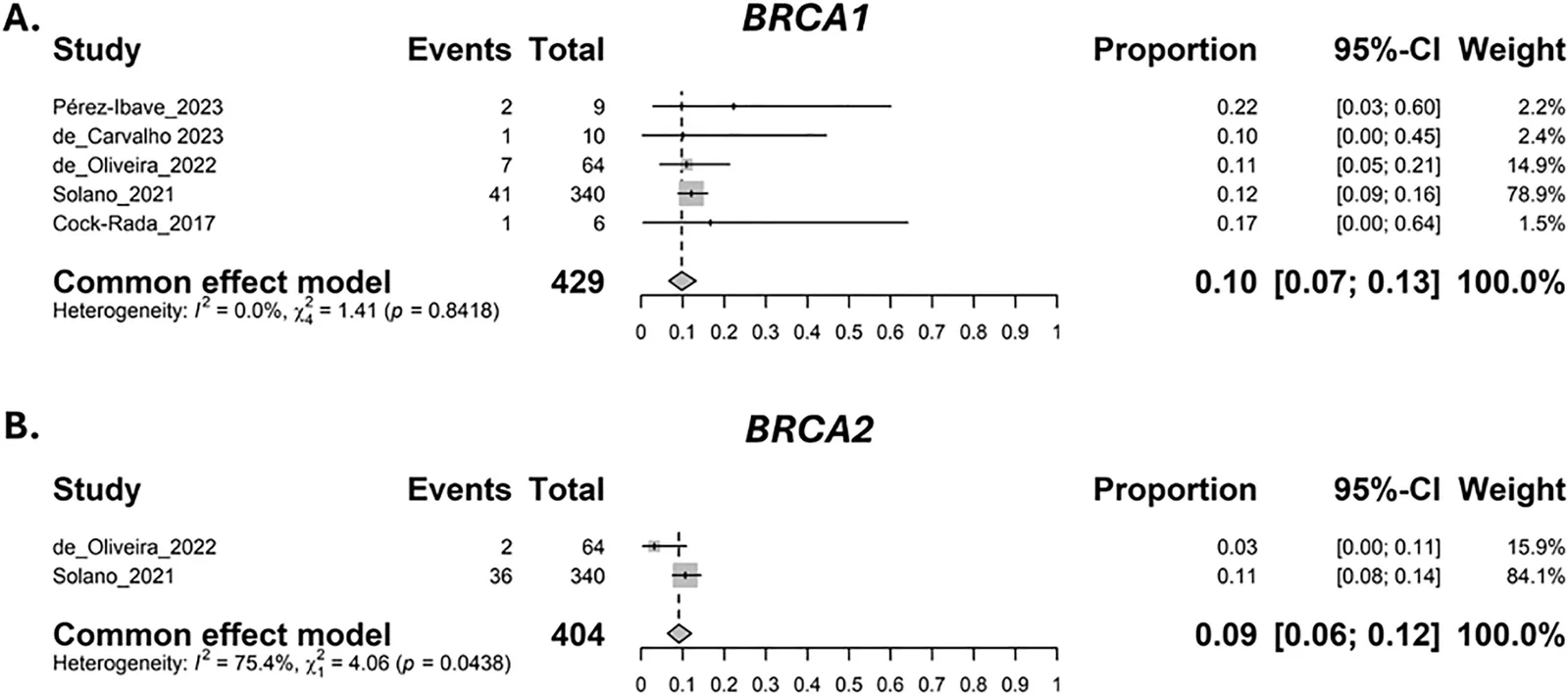
Gene-level prevalence of pathogenic/likely pathogenic germline variants in LATAM ovarian cancer cohorts (A*) BRCA1* common-effect meta-analysis across 5 cohorts (total n=429). (B) *BRCA2* common-effect meta-analysis across 2 cohorts (total n=404).

Vargas-Castellanos. Prevalence and spectrum of variants in homologous recombination repair genes in ovarian cancer in Latin America: a systematic review and meta-analysis. Am J Obstet Gynecol 2026.

## Discussion

This systematic review provides the first synthesis of P and LP variants in HRR pathway genes among ovarian cancer patients in LATAM. We identified 8 studies from 5 countries, all exclusively assessing germline DNA. Although our search strategy was designed to capture both germline and somatic studies, we did not identify any eligible studies evaluating somatic HRR variants within the predefined scope of our review. This finding should be interpreted in the context of our eligibility criteria, which focused on multigene HRR testing and excluded studies restricted exclusively to *BRCA1/2*, whether germline or somatic. This criterion was adopted to extend the assessment beyond the extensively studied *BRCA1/2* genes and to characterize the broader HRR landscape in LATAM, rather than to exclude *BRCA1/2* from the analysis. Indeed, *BRCA1/2* were included when assessed as part of multigene HRR testing and accounted for ∼96% of all identified HRR carriers.

This distinction is clinically relevant, as international guidelines recommend germline testing as the initial step for PARP inhibitor eligibility^18^^,^^59^ but also emphasize somatic testing when germline results are negative or when HRD status can guide the use of PARP inhibitors or platinum-based therapy.^19^^,^^60^ Within the predefined scope of this review, the absence of eligible studies evaluating somatic HRR variants highlights an important evidence gap in the LATAM literature, with potential implications for treatment access and implementation.

Our results highlight the unequal distribution of research and diagnostic capacity across LATAM. Most available data derive from Brazil and Argentina, whereas Chile and Colombia contributed only isolated cohorts, and many countries remain unrepresented.^61^^,^^62^ This concentration of NGS infrastructure in a few centers limits regional representativeness and may exacerbate inequities in access to molecular diagnostics and precision therapies.^63^^,^^64^ These findings underscore the importance of expanding molecular testing capacity and promoting multicenter collaborations to improve equity and ensure broader access to targeted treatments across the region.

Another central finding was the heterogeneity in sequencing approaches. Panels ranged from targeted *BRCA1/2* assays to custom expanded panels and commercial tests, covering anywhere from 25 to 143 genes. To mitigate this variability, we restricted our synthesis to a set of 15 genes most frequently represented in 8 commercial kits and 4 international guidelines: *BRCA1, BRCA2, PALB2, BRIP1, RAD51C, RAD51D, BARD1, ATM, CHEK2, CHEK1, CDK12, NBN, FANCL, RAD51B*, and *RAD54L*. Even so, no global consensus currently exists on which HRR genes should be systematically evaluated.^16^ Our approach suggests that these 15 genes could form the backbone of standardized HRR testing panels for ovarian cancer, ensuring comparability across studies, facilitating prevalence estimation, and supporting clinical decision-making in the region. At the same time, under the new indications in ovarian cancer that increasingly call for HRD rather than HRR testing alone,^8^^,^^65^, ^66^, ^67^ the economic burden of HRD assays compared with NGS-based HRR panels could further hinder implementation in LATAM. This reinforces the need for regionally adapted guidelines and collaborative frameworks to balance cost, access, and clinical relevance.^35^^,^^36^

Variant curation also emerged as a critical consideration. One *CHEK2* variant (c.538C>T, p.Arg180Cys) initially reported as LP was reclassified as VUS after expert review, leading to the exclusion of an entire cohort (Felicio et al.^54^) from our prevalence analyses. This illustrates the importance of periodic reanalysis and expert curation of variants, since misclassification can significantly affect both epidemiological estimates and clinical decisions.^68^^,^^69^

Our meta-analysis estimated a pooled prevalence of germline P/LP HRR variants of 17%, with sensitivity analyses increasing this to 20% after excluding small studies that contributed disproportionately to heterogeneity. Gene specific pooled prevalence was 10% for *BRCA1* and 9% for *BRCA2*, representing nearly 96% of all HRR carriers in LATAM. These estimates are broadly consistent with global reports.^22^^,^^70^, ^71^, ^72^, ^73^, ^74^, ^75^ However, they should be interpreted with caution, as the limited number of studies and methodological variability may underestimate the true variance. The need to restrict gene-level meta-analyses to those assessed in at least 2 cohorts, and to apply common-effect models, reflects the constraints imposed by the available evidence.

When compared with international cohorts, *BRCA1/2* prevalence in LATAM (10% and 9%) was consistent with global estimates, typically ranging between 6% to 12% for *BRCA1* and 3% to 6% for *BRCA2* across Asian and Caucasian populations.^22^^,^^71^^,^^74^, ^75^, ^76^ Non-*BRCA* HRR variants were relatively uncommon (4.2%), a frequency slightly higher than in Asian cohorts (2.6%–5.2%).^71^^,^^74^^,^^76^ While *RAD51D* and *ATM* predominated in Asian studies, *CHEK2* was the most frequent non-*BRCA* gene in LATAM. 2 *CHEK2* P variants were identified in Brazilian patients—c.470T>C and c.485A>G.^54^^,^^55^^,^^57^ The c.470T>C variant is a recognized European founder mutation,^90^ and its presence in LATAM likely reflects historical introduction through colonization. These findings highlight the region’s population-specific genetic background and the limitations imposed by small sample sizes, reinforcing the need for larger, standardized cohorts to clarify the contribution of non-*BRCA* HRR genes.

We identified 5 recurrent variants—*BRCA1* c.211A>G, c.3331_3334del, c.5266dup, and *BRCA2* c.5351dup, c.1909+1G>A—detected in multiple patients and across more than 1 country. For *BRCA1*, c.211A>G was found in Mexico and Argentina^51^^,^^53^ and is a recognized founder mutation in Galicia, Spain, where it accounts for up to 50% of all *BRCA1* P/LP variants in this population.^77^^,^^78^ The c.3331_3334del deletion has been proposed as a founder mutation in Portugal, with haplotype studies confirming shared ancestry.^79^ Its presence in Spain, Africa, Canada, Brazil, and Colombia suggests an Iberian origin before its introduction into South America.^32^^,^^80^^,^^81^

The *BRCA1* c.5266dup (also known as 5382insC or 5385insC) is one of the most widely recognized founder mutations worldwide. First described in Ashkenazi Jewish populations, it also occurs in Slavic and LATAM cohorts.^82^^,^^83^ In Brazil, it has been detected in both Jewish and non-Jewish HBOC families, with higher prevalence among bilateral breast cancer patients.^82^ Haplotype analyses suggest an origin about 1800 years ago in Scandinavia or northern Russia, followed by dissemination across Europe and later into the Ashkenazi and LATAM populations.^84^

For *BRCA2*, the c.5351dup variant was identified in Argentina^53^ and has also been reported in Ashkenazi Jewish populations.^85^ The splice-site variant c.1909+1G>A occurred at relatively high frequency in the Argentinian cohort but has not been described in large international series, limiting current knowledge of its distribution and clinical relevance. Further investigation is needed to determine whether c.1909+1G>A may represent a founder effect in LATAM populations. Overall, the recurrence of these variants across countries and ethnic backgrounds suggests that some mutations were introduced during colonization and subsequently expanded locally, indicating their potential utility as first-line markers in cost-effective screening panels for the region.

The 3 variants in *CHEK2* (c.470T>C; c.538C>T; c.485A>G) gene were identified in Brazil.^54^^,^^55^^,^^57^ The variant *CHEK2* c.470T>C was identified in a study of 1682 individuals who underwent genotyping with panels of 27 to 78 genes to detect germ variants in genes predisposed to cancer. This variant was described in a patient with ovarian cancer with a family history of breast cancer.^57^ Studies in the Caucasian population reported this variant in patients with thyroid cancer describing that those carrying this variant had a double risk of developing differentiated thyroid cancer.^86^ Similarly, Švajdler et al. studied patients with granuloses cell tumors, finding that the presence of the variant increased the risk of this neoplasm.^87^ This variant is classified as a more common European founder mutation of *CHEK2*.^87^ The identification of this variant in Latin American populations suggests that it was probably introduced during the colonization process, which would explain its detection in both Caucasian and Latin American populations. This finding highlights the importance of population genetics studies to understand the distribution of founding variants and their clinical impact, especially in the context of hereditary ovarian cancer.

Several limitations should be noted. Cohort sizes were small, ancestry and clinical variables such as family history were inconsistently reported, and 2 studies did not provide age at diagnosis. Methodological heterogeneity was also substantial, encompassing differences in panel composition, sequencing platforms, and reporting standards. The lack of functional validation and the need for periodic variant reclassification, exemplified by the *CHEK2* case, further complicate interpretation. Moreover, by restricting the analysis to variants classified as P/LP in ClinVar or in the original reports, emerging VUS that may warrant functional evaluation were not considered.

Between-study heterogeneity (I²=56.2%, 95% CI: 0%–81%) reflects sparse-data effects rather than true clinical or methodological diversity. This inconsistency was resolved by removing 2 micro-cohorts (n=1 and 3), and a sparse binomial model estimated zero between-study variance. Subgroup analyses were underpowered to exclude meaningful clinical effects, and meta-regression was unfeasible. Furthermore, sequencing platform was missing in 2 cohorts, and histological subtype—a key *BRCA1/2* determinant—was unreported across all studies. Consequently, limited cohorts prevented variance estimation, meaning gene-level estimates relied on common-effect models strictly as descriptive summaries.

Regarding ascertainment bias, an Argentinian cohort contributes 77% of the pooled sample; notably, all its carriers had a family history of cancer. Although labeled “hospital-based,” it effectively represents a high-risk referral population. Because this cohort dominates our analysis, the summary prevalence applies specifically to Latin American women undergoing genetic testing due to familial risk or early-onset cancer, rather than unselected ovarian cancer populations. This discrepancy between nominal recruitment labels and actual clinical selection explains why comparisons between hereditary-clinic and hospital-based cohorts proved uninformative. Furthermore, countries with limited or no representation in the current evidence base may have different genetic backgrounds, founder variants, patterns of hereditary cancer referral, access to genetic testing, and clinical selection criteria. Further multicenter studies incorporating more geographically diverse LATAM populations are needed to determine whether the observed HRR variant spectrum and prevalence are consistent across the region.

Histological subtypes are an important determinant when interpreting the prevalence and clinical implications of HRD in ovarian cancer. Previous studies have shown that HRD is more frequently observed in serous carcinomas compared with other histological subtypes. Elvin et al.^88^ reported HRD, defined by *BRCA* mutation and/or high loss of heterozygosity (LOH), in 43.8% of serous carcinomas, compared with 37.6% of endometrioid carcinomas, 23.5% of carcinosarcomas, 13.6% of clear cell carcinomas, and 8.1% of mucinous tumors. Other studies assessing HRR gene mutations similarly reported frequencies of 27% to 31% in serous and 23.8% to 27% in endometrioid carcinomas.^6^^,^^89^ Furthermore, within high-grade serous ovarian carcinoma, HRD status has been associated with specific morphological patterns. Hanna et al.^90^ reported enrichment of solid, pseudo-endometrioid, and transitional-like morphology in *BRCA1* and *BRCA2* mutated tumors (57% and 67%, respectively), compared with *BRCA1/2*-wild-type HRD (38%) and homologous recombination-proficient tumors (18%; *P*=.019). These differences have important therapeutic implications, as HRD tumors are particularly sensitive to DNA-damaging platinum-based chemotherapy and to PARP inhibitors. The therapeutic effect of PARP inhibitors is based on synthetic lethality resulting from simultaneous impairment of homologous recombination and PARP-mediated DNA repair. Although the greatest benefit is generally observed in *BRCA1/2*-mutated tumors, responses are also observed in *BRCA*-wild-type HRD tumors, supporting HRD assessment beyond *BRCA* mutation status alone.^91^

Despite these limitations, our findings highlight both opportunities and challenges for the integration of HRR and HRD testing into clinical practice in LATAM. The predominance of *BRCA1/2* mutations supports the priority of germline testing for hereditary risk assessment and therapy selection, while the absence of somatic and HRD studies underscores important gaps that could restrict patient access to PARP inhibitors and precision therapies. Structural barriers including methodological heterogeneity, limited research capacity in many countries, and the need for standardized reclassification frameworks must be addressed. Moving forward, regionally adapted guidelines, expanded NGS infrastructure, and strengthened collaborative networks will be essential to harmonize testing practices, ensure cost-effective implementation, and promote equitable access to molecularly guided care for ovarian cancer patients across the region.

## Data availability

The data underlying this article are available in the article and in its online supplementary material.

## CRediT authorship contribution statement

**Elizabeth Vargas-Castellanos:** Writing – review & editing, Writing – original draft, Supervision, Methodology, Investigation, Formal analysis, Conceptualization. **Natalia Santiago-Tovar:** Writing – original draft, Data curation. **Luisa Murcia-Soriano:** Writing – original draft, Data curation. **Ariana Patino-Herrera:** Writing – original draft, Data curation. **Rafael Parra-Medina:** Writing – original draft, Data curation. **Julian C. Riano-Moreno:** Writing – review & editing, Writing – original draft, Formal analysis, Conceptualization.

## Appendix Supplementary materials

Image, application 1

## Footnotes

Supplementary material associated with this article can be found in the online version at doi:10.1016/j.xagr.2026.100693.
