# Supplementary material for: Prevalence and spectrum of variants in homologous recombination repair genes in ovarian cancer in Latin America: a systematic review and meta-analysis
Source: AJOG Glob Rep. 2026 Sep 12;6(4):100693. doi: 10.1016/j.xagr.2026.100693 (PMC13679366; doi:10.1016/j.xagr.2026.100693)
Supplement: Supplementary file 1 [file mmc1.docx]

| **Test (Manufacturer)** | **Sample / Type** | **Regulatory Status** | **HRD / GIS Reporting** | **Number HRR/related genes** | **Genes covered (HRR-related)** | **Note** | **Ref** |
| --- | --- | --- | --- | --- | --- | --- | --- |
| FoundationOne® CDx (FMI) | Tumor (FFPE, CGP) | FDA PMA (multi-drug CDx) | No HRD score | 14 | *ATM, BRCA1, BRCA2, BARD1, BRIP1, CDK12, CHEK1, CHEK2, FANCL, PALB2, RAD51B, RAD51C, RAD51D, RAD54L* | Approved as CDx for PARP inhibitors and targeted therapies. | (39) |
| myChoice® CDx / CDx Plus (Myriad) | Tumor (FFPE) | FDA PMA (HRD in ovarian; BRCA1/2 CDx) | Yes: GIS (0–100) | 15 | *ATM, BARD1, BRCA1, BRCA2, BRIP1, CDK12, CHEK1, CHEK2, FANCL, PALB2, PPP2R2A, RAD51B, RAD51C, RAD51D, RAD54L* | Reports GIS plus BRCA1/2 variants including large rearrangements. | (40) |
| NeoTYPE® HRR Profile (NeoGenomics) | Tumor NGS + IHC | LDT (US) | No HRD score | 31 | *ATM, ATR, BARD1, BRCA1, BRCA2, BRIP1, CDK12, CHEK1, CHEK2, FANCA, FANCC, FANCD2, FANCE, FANCF, FANCG, FANCL, MLH1, MRE11A, MSH2, MSH6, NBN, PALB2, PMS2, RAD50, RAD51, RAD51B, RAD51C, RAD51D, RAD54L, TP53* | Broad HRR coverage; includes Fanconi anemia and MMR genes. | (41) |
| SOPHiA DDM™ Dx HRD Solution (CE-IVD) | Tumor (capture + low-pass WGS) | CE-IVD (EU) | Yes: HRD algorithm | 28 | *ATM, BARD1, BRCA1, BRCA2, BRIP1, CDK12, CHEK1, CHEK2, FANCA, FANCD2, FANCL, MRE11, NBN, PALB2, PPP2R2A, PTEN, RAD51B, RAD51C, RAD51D, RAD54L, TP53* | Combines HRR variant panel with HRD genomic scars. | (42) |
| OncoDEEP® (OncoDNA) | Tumor CGP (≈638 genes) | CE-IVD / LDT | No HRD score | Subset | *ATM, ATR, BRCA1, BRCA2, BRIP1, CDK12, CHEK1, CHEK2, NBN, PALB2, ARID1A, BARD1* | Large panel; HRR subset shown. | (43) |
| Invitae Hereditary Breast & Gyn Cancers | Germline (blood/saliva) | LDT | No HRD score (risk only) | 20 | *ATM, BARD1, BRCA1, BRCA2, BRIP1, CDH1, CHEK2, EPCAM, MLH1, MSH2, MSH6, NF1, PALB2, PMS2, PTEN, RAD51C, RAD51D, STK11, TP53, NBN* | Hereditary risk; not CDx. | (44) |
| BRACAnalysis® CDx (Myriad) | Germline (blood) | FDA PMA (gBRCA CDx) | No HRD score | 2 | *BRCA1, BRCA2* | Guides PARPi therapy in gBRCA carriers. | (45) |
| TruSight™ Oncology 500 HRD (Illumina + Myriad) | Tumor CGP | CE-IVD (EU); research use in US | Yes: GIS (Myriad) | 23+ | *ATM, ATR, BARD1, BRCA1, BRCA2, BRIP1, CDK12, CHEK1, CHEK2, FANCA, FANCC, FANCI, FANCL, NBN, PALB2, RAD50, RAD51, RAD51B, RAD51C, RAD51D, RAD54L, TP53, PTEN* | Comprehensive CGP + GIS integration. | (46) |

**Supplemental Table 1. Commercial CDx and HRR-related assays covering BRCA1/2 and non-BRCA genes.** Overview of commercially available companion diagnostic (CDx) assays and clinical-grade panels assessing homologous recombination repair (HRR) genes. FFPE, formalin-fixed paraffin-embedded; CGP, comprehensive genomic profiling; FDA PMA, U.S. Food and Drug Administration premarket approval; CDx, companion diagnostic; HRD, homologous recombination deficiency; GIS, genomic instability score; CE-IVD, Conformité Européenne in vitro diagnostic; LDT, laboratory-developed test; WGS, whole-genome sequencing; g*BRCA*, germline *BRCA*.

| **Guideline** | **Genes** | **Comment or recommendation** | **Ref** |
| --- | --- | --- | --- |
| ESMO recommendations | *BRCA1, BRCA2, PALB2, RAD51, RAD51C, RAD51D* | Strong for *BRCA1/2*; emerging: *RAD51, RAD51C/D, PALB2.* | (18) |
| European Consensus | *BARD1, BRCA1, BRCA2, BRIP1, PALB2, RAD51C, RAD51D* | BRCA1/2 recommended; non‑BRCA (*RAD51C/D, BRIP1, PALB2, BARD1*) considered for familial risk; not predictive of PARPi in 1st line. | (47) |
| NCCN Guidelines 2026 (Hereditary Cancer Testing) | *ATM, BRCA1, BRCA2, BRIP1, PALB2, RAD51C, RAD51D* | Lists *ATM, BRCA1/2, BRIP1, PALB2, RAD51C/D* as ovarian susceptibility genes. | (48) |
| German Ovarian Cancer Commission 2025 | *BARD1, BRCA1, BRCA2, BRIP1, CHEK1, CHEK2, FAM175A, NBN, PALB2, RAD51C, RAD51D* | Includes *BRCA1/2* and other HRR (*BARD1, BRIP1, CHEK1/2, FAM175A, NBN, PALB2, RAD51C/D*). | (29) |

**Supplemental Table 2. HRR-related genes recommended/considered by international guidelines for ovarian cancer**

| **Gene** | **Count (Panels + Guidelines)** |
| --- | --- |
| *BRCA1* | 12 |
| *BRCA2* | 12 |
| *PALB2* | 11 |
| *BRIP1* | 10 |
| *RAD51C* | 10 |
| *RAD51D* | 10 |
| *BARD1* | 9 |
| *ATM* | 8 |
| *CHEK2* | 8 |
| *CHEK1* | 7 |
| *CDK12* | 6 |
| *NBN* | 6 |
| *FANCL* | 5 |
| *RAD51B* | 5 |
| *RAD54L* | 5 |
| *TP53* | 4 |
| *ATR* | 3 |
| *FANCA* | 3 |
| *PTEN* | 3 |
| *RAD51* | 3 |

**Supplemental Table 3. Count of HRR genes across commercial panels and international guidelines.** Genes appearing five times or more across these sources were included in our study (gray shading).

| **Database** | **Search Strategy** |
| --- | --- |
| Ovid MEDLINE | 1 exp Ovarian Neoplasms/ 2 (Ovary adj2 Cancer Patient*).mp. 3 (Ovarian adj2 Cancer).mp. 4 (Ovary adj2 Cancer).mp. 5 1 or 2 or 3 or 4  6 ATM.mp. 7 BRIP1.mp. 8 BARD1.mp. 9 CDK12.mp. 10 CHEK1.mp. 11 CHEK2.mp. 12 FANCL.mp. 13 PALB2.mp. 14 RAD51B.mp. 15 RAD51C.mp. 16 RAD51D.mp. 17 RAD54L.mp. 18 BRCA1.mp. 19 BRCA2.mp. 20 NBN.mp.  21 or/6-20  22 HRR.mp. 23 HRRm.mp. 24 ((HRR adj1 gene*) or (HRR adj1 genes*)).ti,ab. 25 ("Homologous recombination repair" adj2 gene*).ti,ab. 26 Recombinational DNA Repair/ 27 (("Homologous recombination" or somatic or Germline) and ("gene panel" or "panel test" or "multi-gene panel test")).ti,ab. 28 ("Panel sequenc*" adj2 gene*).ti,ab.  29 5 and 21 and (22 or 23 or 24 or 25 or 26 or 27 or 28)  30 Hispanic.mp. 31 Central America/ or South America/ 32 Latin America/ 33 ("Costa Rica" or "El Salvador" or "Guatemala" or "Honduras" or "Nicaragua" or "Panamá" or "Panama" or "Argentina" or "Bolivia" or "Brasil" or "Brazil" or "Chile" or "Colombia" or "Ecuador" or "Guyana" or "Paraguay" or "Perú" or "Peru" or "Uruguay" or "Venezuela").ab,ti. 34 30 or 31 or 32 or 33 35 29 and 34 |
| Embase | #1 'ovarian tumor'/exp OR 'ovarian tumor' #2 ovary:ab,ti AND cancer:ab,ti AND patient*:ab,ti #3 'ovary cancer':ab,ti #4 'ovarian cancer':ab,ti #5 #1 OR #2 OR #3 OR #4  #6 'atm gene':ab,ti #7 'brip1 gene':ab,ti #8 'bard1 gene':ab,ti #9 'cdk12 gene':ab,ti #10 'chek1 gene':ab,ti #11 'chek2 gene':ab,ti #12 'fancl gene':ab,ti #13 'palb2 gene':ab,ti #14 'rad51b gene':ab,ti #15 'rad51c gene':ab,ti #16 'rad51d gene':ab,ti #17 'rad54l gene':ab,ti #18 'brca1 gene':ab,ti #19 'brca2 gene':ab,ti #20 'nbn gene':ab,ti  #21 hrr:ab,ti #22 hrrm:ab,ti #23 hrr AND gene OR (hrr:ti,ab AND genes:ti,ab) #24 homologous:ti,ab AND recombination:ti,ab AND repair:ti,ab AND gene:ti,ab #25 'homologous recombination'/exp #26 ('homologous recombination':ti,ab OR somatic:ti,ab OR germline:ti,ab) AND ('gene panel':ti,ab OR 'panel test':ti,ab OR 'multi-gene panel test') #27 'panel sequenc*':ti,ab AND gene*:ti,ab  #28 #6 OR #7 OR #8 OR #9 OR #10 OR #11 OR #12 OR #13 OR #14 OR #15 OR #16 OR #17 OR #18 OR #19 OR #20 #29 #21 OR #22 OR #23 OR #24 OR #25 OR #26 OR #27  #30 'hispanic':ab,ti #31 'central america'/exp OR 'south america'/exp #32 'latin america'/exp #33 'costa rica':ti,ab OR 'el salvador':ti,ab OR 'guatemala':ti,ab OR 'honduras':ti,ab OR 'nicaragua':ti,ab OR 'panamá':ti,ab OR 'panama':ti,ab OR 'argentina':ti,ab OR 'bolivia':ti,ab OR 'brasil':ti,ab OR 'brazil':ti,ab OR 'chile':ti,ab OR 'colombia':ti,ab OR 'ecuador':ti,ab OR 'guyana':ti,ab OR 'paraguay':ti,ab OR 'perú':ti,ab OR 'peru':ti,ab OR 'uruguay':ti,ab OR 'venezuela':ti,ab  #34 #30 OR #31 OR #32 OR #33  #35 #5 AND (#28 OR #29) AND #34 |
| Cochrane | #1 MeSH descriptor: [Ovarian Neoplasms] explode all trees #2 Ovary Cancer Patient #3 Ovarian Cancer #4 Ovary Cancer #5 #1 OR #2 OR #3 OR #4  #6 'atm gene' #7 'brip1 gene' #8 'bard1 gene' #9 'cdk12 gene' #10 'chek1 gene' #11 'chek2 gene' #12 'fancl gene' #13 'palb2 gene' #14 'rad51b gene' #15 'rad51c gene' #16 'rad51d gene' #17 'rad54l gene' #18 'brca1 gene' #19 'brca2 gene' #20 'nbn gene'  #21 hrr #22 hrrm #23 (hrr AND gene) OR (hrr AND genes) #24 homologous AND recombination #25 MeSH descriptor: [Homologous Recombination] explode all trees #26 ('homologous recombination' OR somatic OR germline) AND ('gene panel' OR 'panel test' OR 'multi-gene panel test') #27 'panel sequenc*' AND gene*  #28 #6 OR #7 OR #8 OR #9 OR #10 OR #11 OR #12 OR #13 OR #14 OR #15 OR #16 OR #17 OR #18 OR #19 OR #20 OR #21 OR #22 OR #23 OR #24 OR #25 OR #26 OR #27  #29 MeSH descriptor: [Hispanic or Latino] explode all trees #30 MeSH descriptor: [Central America] explode all trees #31 MeSH descriptor: [South America] explode all trees #32 MeSH descriptor: [Latin America] explode all trees #33 'costa rica' OR 'el salvador' OR 'guatemala' OR 'honduras' OR 'nicaragua' OR 'panamá' OR 'panama' OR 'argentina' OR 'bolivia' OR 'brasil' OR 'brazil' OR 'chile' OR 'colombia' OR 'ecuador' OR 'guyana' OR 'paraguay' OR 'perú' OR 'peru' OR 'uruguay' OR 'venezuela'  #34 #29 OR #30 OR #31 OR #32 OR #33  #35 #5 AND #28 AND #34 |
| LILACS | ((Ovarian Neoplasms) OR (Ovary Cancer Patient) OR (Ovarian Cancer) OR (Ovary Cancer)) AND  ((hrr) OR (hrr gene) OR (Homologous Recombination) OR (gene panel) OR (multi-gene panel test)  OR ('atm gene') OR ('brip1 gene') OR ('bard1 gene') OR ('cdk12 gene') OR ('chek1 gene') OR ('chek2 gene')  OR ('fancl gene') OR ('palb2 gene') OR ('rad51b gene') OR ('rad51c gene') OR ('rad51d gene')  OR ('rad54l gene') OR ('brca2 gene') OR ('brca1 gene') OR ('nbn gene')) AND  ((Hispanic or Latino) OR (Central America) OR (South America) OR (Latin America)  OR ('costa rica' OR 'el salvador' OR 'guatemala' OR 'honduras' OR 'nicaragua' OR 'panamá' OR 'panama'  OR 'argentina' OR 'bolivia' OR 'brasil' OR 'brazil' OR 'chile' OR 'colombia' OR 'ecuador' OR 'guyana'  OR 'paraguay' OR 'perú' OR 'peru' OR 'uruguay' OR 'venezuela'))) |
| Epistemonikos | (title:((Ovarian Neoplasms) OR (Ovary Cancer Patient) OR (Ovarian Cancer) OR (Ovary Cancer)) OR abstract:((Ovarian Neoplasms) OR (Ovary Cancer Patient) OR (Ovarian Cancer) OR (Ovary Cancer))) AND (title:((hrr) OR (hrr gene) OR (Homologous Recombination) OR (gene panel) OR (multi-gene panel test)  OR ('atm gene') OR ('brip1 gene') OR ('bard1 gene') OR ('cdk12 gene') OR ('chek1 gene') OR ('chek2 gene')  OR ('fancl gene') OR ('palb2 gene') OR ('rad51b gene') OR ('rad51c gene') OR ('rad51d gene')  OR ('rad54l gene') OR ('brca2 gene') OR ('brca1 gene') OR ('nbn gene')))  OR abstract:((hrr) OR (hrr gene) OR (Homologous Recombination) OR (gene panel) OR (multi-gene panel test)  OR ('atm gene') OR ('brip1 gene') OR ('bard1 gene') OR ('cdk12 gene') OR ('chek1 gene') OR ('chek2 gene')  OR ('fancl gene') OR ('palb2 gene') OR ('rad51b gene') OR ('rad51c gene') OR ('rad51d gene')  OR ('rad54l gene') OR ('brca2 gene') OR ('brca1 gene') OR ('nbn gene'))) AND  (title:((Hispanic OR Latino) OR (Central America) OR (South America) OR (Latin America) OR ('costa rica' OR 'el salvador' OR 'guatemala' OR 'honduras' OR 'nicaragua' OR 'panamá' OR 'panama' OR 'argentina' OR 'bolivia' OR 'brasil' OR 'brazil' OR 'chile' OR 'colombia' OR 'ecuador' OR 'guyana' OR 'paraguay' OR 'perú' OR 'peru' OR 'uruguay' OR 'venezuela'))  OR abstract:((Hispanic OR Latino) OR (Central America) OR (South America) OR (Latin America) OR ('costa rica' OR 'el salvador' OR 'guatemala' OR 'honduras' OR 'nicaragua' OR 'panamá' OR 'panama' OR 'argentina' OR 'bolivia' OR 'brasil' OR 'brazil' OR 'chile' OR 'colombia' OR 'ecuador' OR 'guyana' OR 'paraguay' OR 'perú' OR 'peru' OR 'uruguay' OR 'venezuela')) ) |
| Scopus | ( TITLE-ABS-KEY ( ( ovarian AND neoplasms ) OR ( ovary AND cancer AND patient ) OR ( ovarian AND cancer ) OR ( ovary AND cancer ) ) AND  TITLE-ABS-KEY ( ( hrr ) OR ( hrr AND gene ) OR ( homologous AND recombination ) OR ( gene AND panel ) OR ( multi-gene AND panel AND test )  OR ( 'atm AND gene' ) OR ( 'brip1 AND gene' ) OR ( 'bard1 AND gene' ) OR ( 'cdk12 AND gene' ) OR ( 'chek1 AND gene' ) OR ( 'chek2 AND gene' )  OR ( 'fancl AND gene' ) OR ( 'palb2 AND gene' ) OR ( 'rad51b AND gene' ) OR ( 'rad51c AND gene' ) OR ( 'rad51d AND gene' )  OR ( 'rad54l AND gene' ) OR ( 'brca2 AND gene' ) OR ( 'brca1 AND gene' ) OR ( 'nbn AND gene' ) ) AND TITLE-ABS-KEY ( ( hispanic OR latino ) OR ( central AND america ) OR ( south AND america ) OR ( latin AND america )  OR ( 'costa AND rica' OR 'el AND salvador' OR 'guatemala' OR 'honduras' OR 'nicaragua' OR 'panamá' OR 'panama' OR 'argentina' OR 'bolivia'  OR 'brasil' OR 'brazil' OR 'chile' OR 'colombia' OR 'ecuador' OR 'guyana' OR 'paraguay' OR 'perú' OR 'peru' OR 'uruguay' OR 'venezuela' ) ) ) |
| Web of Science | (Ovarian Neoplasms OR Ovary Cancer Patient OR Ovarian Cancer OR Ovary Cancer) (Abstract) AND  (hrr OR hrr gene OR Homologous Recombination OR gene panel OR multi-gene panel test  OR 'atm gene' OR 'brip1 gene' OR 'bard1 gene' OR 'cdk12 gene' OR 'chek1 gene' OR 'chek2 gene'  OR 'fancl gene' OR 'palb2 gene' OR 'rad51b gene' OR 'rad51c gene' OR 'rad51d gene'  OR 'rad54l gene' OR 'brca2 gene' OR 'brca1 gene' OR 'nbn gene') (Abstract) AND  (Hispanic OR Latino OR Central America OR South America OR Latin America  OR 'costa rica' OR 'el salvador' OR 'guatemala' OR 'honduras' OR 'nicaragua' OR 'panamá' OR 'panama'  OR 'argentina' OR 'bolivia' OR 'brasil' OR 'brazil' OR 'chile' OR 'colombia' OR 'ecuador' OR 'guyana' OR 'paraguay' OR 'perú' OR 'peru' OR 'uruguay' OR 'venezuela') (Abstract) |

**Supplemental Table 4. Adjusted search strategies by database for homologous recombination repair (HRR) genes in ovarian cancer.**

| **Database** | **Records identified** |
| --- | --- |
| Medline (Ovid) | 404 |
| Embase (Ovid) | 129 |
| Scopus | 123 |
| Web Of Sciences | 16 |
| LILACS | 15 |
| Cochrane | 12 |
| Epistemonikos | 4 |

**Supplemental Table 5. Records identified per database in the systematic search for HRR genes in ovarian cancer.**

| **study_id (ref)** | **Country** | **ID_case** | **Age_dx** | **Familial_history_cancer** | **ancestry_major** |
| --- | --- | --- | --- | --- | --- |
| Pérez-Ibave et al., 2023 (51) | Mexico | 1_1_BRCA1 | 38 | NR | Hispanic, LATAM/caribbean, European, African, Northamerica descent |
|  |  | 1_2_BRCA1 | 34 | NR | Hispanic, LATAM/caribbean, European, African, Northamerica descent |
| De Carvalho et al., 2023 (52) | Brazil | 2_1_BRCA1 | 36 | NR | LATAM/caribbean,European descent |
| De Oliveira et al., 2022 (57) | Brazil | 3_1_BRCA2 | NR | thyroid/parathyroid, gastric | LATAM/caribbean,European descent |
|  |  | 3_2_BRCA2 | NR | gastric | LATAM/caribbean,European descent |
|  |  | 3_3_BRCA1 | NR | breast | LATAM/caribbean,European descent |
|  |  | 3_4_BRCA1 | NR | prostate | LATAM/caribbean,European descent |
|  |  | 3_5_BRCA1 | NR | skin (non-melanoma) | LATAM/caribbean,European descent |
|  |  | 3_6_BRCA1 | NR | breast, prostate | LATAM/caribbean,European descent |
|  |  | 3_7_BRCA1 | NR | breast | LATAM/caribbean,European descent |
|  |  | 3_8_BRCA1 | NR | breast, colorectal, uterus, pancreas | LATAM/caribbean,European descent |
|  |  | 3_9_BRCA1 | NR | breast | LATAM/caribbean,European descent |
|  |  | 3_10_CHEK2 | NR | breast | LATAM/caribbean,European descent |
|  |  | 3_11_BARD1 | NR | breast, ovarian, uterus cancer | LATAM/caribbean,European descent |
| Solano et al., 2021  (53) | Argentina | 4_1_BRCA2 | 61 | present | Asian, European, African, LATAM/caribbean, Northamerica descent |
|  |  | 4_2_BRCA2 | 55 | present | Asian, European, African, LATAM/caribbean, Northamerica descent |
|  |  | 4_3_BRCA2 | 67 | present | Asian, European, African, LATAM/caribbean, Northamerica descent |
|  |  | 4_4_BRCA2 | 66 | present | Asian, European, African, LATAM/caribbean, Northamerica descent |
|  |  | 4_5_BRCA2 | 50 | present | Asian, European, African, LATAM/caribbean, Northamerica descent |
|  |  | 4_6_BRCA2 | 59 | present | Asian, European, African, LATAM/caribbean, Northamerica descent |
|  |  | 4_7_BRCA2 | 63 | present | Asian, European, African, LATAM/caribbean, Northamerica descent |
|  |  | 4_8_BRCA2 | 54 | present | Asian, European, African, LATAM/caribbean, Northamerica descent |
|  |  | 4_9_BRCA2 | 55 | present | Asian, European, African, LATAM/caribbean, Northamerica descent |
|  |  | 4_10_BRCA2 | 63 | present | Asian, European, African, LATAM/caribbean, Northamerica descent |
|  |  | 4_11_BRCA2 | 54 | present | Asian, European, African, LATAM/caribbean, Northamerica descent |
|  |  | 4_12_BRCA2 | 66 | present | Asian, European, African, LATAM/caribbean, Northamerica descent |
|  |  | 4_13_BRCA2 | 53 | present | Asian, European, African, LATAM/caribbean, Northamerica descent |
|  |  | 4_14_BRCA2 | 58 | present | Asian, European, African, LATAM/caribbean, Northamerica descent |
|  |  | 4_15_BRCA2 | 58 | present | Asian, European, African, LATAM/caribbean, Northamerica descent |
|  |  | 4_16_BRCA2 | 56 | present | Asian, European, African, LATAM/caribbean, Northamerica descent |
|  |  | 4_17_BRCA2 | 60 | present | Asian, European, African, LATAM/caribbean, Northamerica descent |
|  |  | 4_18_BRCA2 | 59 | present | Asian, European, African, LATAM/caribbean, Northamerica descent |
|  |  | 4_19_BRCA2 | 62 | present | Asian, European, African, LATAM/caribbean, Northamerica descent |
|  |  | 4_20_BRCA2 | 58 | present | Asian, European, African, LATAM/caribbean, Northamerica descent |
|  |  | 4_21_BRCA2 | 74 | present | Asian, European, African, LATAM/caribbean, Northamerica descent |
|  |  | 4_22_BRCA2 | 50 | present | Asian, European, African, LATAM/caribbean, Northamerica descent |
|  |  | 4_23_BRCA2 | 51 | present | Asian, European, African, LATAM/caribbean, Northamerica descent |
|  |  | 4_24_BRCA2 | 59 | present | Asian, European, African, LATAM/caribbean, Northamerica descent |
|  |  | 4_25_BRCA2 | 61 | present | Asian, European, African, LATAM/caribbean, Northamerica descent |
|  |  | 4_26_BRCA2 | 66 | present | Asian, European, African, LATAM/caribbean, Northamerica descent |
|  |  | 4_27_BRCA2 | 45 | present | Asian, European, African, LATAM/caribbean, Northamerica descent |
|  |  | 4_28_BRCA2 | 49 | present | Asian, European, African, LATAM/caribbean, Northamerica descent |
|  |  | 4_29_BRCA2 | 49 | present | Asian, European, African, LATAM/caribbean, Northamerica descent |
|  |  | 4_30_BRCA2 | 51 | present | Asian, European, African, LATAM/caribbean, Northamerica descent |
|  |  | 4_31_BRCA2 | 55 | present | Asian, European, African, LATAM/caribbean, Northamerica descent |
|  |  | 4_32_BRCA2 | 50 | present | Asian, European, African, LATAM/caribbean, Northamerica descent |
|  |  | 4_33_BRCA2 | 40 | present | Asian, European, African, LATAM/caribbean, Northamerica descent |
|  |  | 4_34_BRCA2 | 49 | present | Asian, European, African, LATAM/caribbean, Northamerica descent |
|  |  | 4_35_BRCA2 | 56 | present | Asian, European, African, LATAM/caribbean, Northamerica descent |
|  |  | 4_36_BRCA2 | 60 | present | Asian, European, African, LATAM/caribbean, Northamerica descent |
|  |  | 4_36_BRCA1 | 33 | present | Asian, European, African, LATAM/caribbean, Northamerica descent |
|  |  | 4_37_BRCA1 | 41 | present | Asian, European, African, LATAM/caribbean, Northamerica descent |
|  |  | 4_38_BRCA1 | 51 | present | Asian, European, African, LATAM/caribbean, Northamerica descent |
|  |  | 4_39_BRCA1 | 77 | present | Asian, European, African, LATAM/caribbean, Northamerica descent |
|  |  | 4_40_BRCA1 | 45 | present | Asian, European, African, LATAM/caribbean, Northamerica descent |
|  |  | 4_41_BRCA1 | 68 | present | Asian, European, African, LATAM/caribbean, Northamerica descent |
|  |  | 4_42_BRCA1 | 59 | present | Asian, European, African, LATAM/caribbean, Northamerica descent |
|  |  | 4_43_BRCA1 | 56 | present | Asian, European, African, LATAM/caribbean, Northamerica descent |
|  |  | 4_44_BRCA1 | 49 | present | Asian, European, African, LATAM/caribbean, Northamerica descent |
|  |  | 4_45_BRCA1 | 50 | present | Asian, European, African, LATAM/caribbean, Northamerica descent |
|  |  | 4_46_BRCA1 | 59 | present | Asian, European, African, LATAM/caribbean, Northamerica descent |
|  |  | 4_47_BRCA1 | 42 | present | Asian, European, African, LATAM/caribbean, Northamerica descent |
|  |  | 4_48_BRCA1 | 70 | present | Asian, European, African, LATAM/caribbean, Northamerica descent |
|  |  | 4_49_BRCA1 | 65 | present | Asian, European, African, LATAM/caribbean, Northamerica descent |
|  |  | 4_50_BRCA1 | 55 | present | Asian, European, African, LATAM/caribbean, Northamerica descent |
|  |  | 4_51_BRCA1 | 55 | present | Asian, European, African, LATAM/caribbean, Northamerica descent |
|  |  | 4_52_BRCA1 | 49 | present | Asian, European, African, LATAM/caribbean, Northamerica descent |
|  |  | 4_53_BRCA1 | 49 | present | Asian, European, African, LATAM/caribbean, Northamerica descent |
|  |  | 4_54_BRCA1 | 57 | present | Asian, European, African, LATAM/caribbean, Northamerica descent |
|  |  | 4_55_BRCA1 | 48 | present | Asian, European, African, LATAM/caribbean, Northamerica descent |
|  |  | 4_56_BRCA1 | 61 | present | Asian, European, African, LATAM/caribbean, Northamerica descent |
|  |  | 4_57_BRCA1 | 49 | present | Asian, European, African, LATAM/caribbean, Northamerica descent |
|  |  | 4_58_BRCA1 | 46 | present | Asian, European, African, LATAM/caribbean, Northamerica descent |
|  |  | 4_59_BRCA1 | 39 | present | Asian, European, African, LATAM/caribbean, Northamerica descent |
|  |  | 4_60_BRCA1 | 65 | present | Asian, European, African, LATAM/caribbean, Northamerica descent |
|  |  | 4_61_BRCA1 | 59 | present | Asian, European, African, LATAM/caribbean, Northamerica descent |
|  |  | 4_62_BRCA1 | 35 | present | Asian, European, African, LATAM/caribbean, Northamerica descent |
|  |  | 4_63_BRCA1 | 50 | present | Asian, European, African, LATAM/caribbean, Northamerica descent |
|  |  | 4_64_BRCA1 | 42 | present | Asian, European, African, LATAM/caribbean, Northamerica descent |
|  |  | 4_65_BRCA1 | 48 | present | Asian, European, African, LATAM/caribbean, Northamerica descent |
|  |  | 4_66_BRCA1 | 72 | present | Asian, European, African, LATAM/caribbean, Northamerica descent |
|  |  | 4_67_BRCA1 | 52 | present | Asian, European, African, LATAM/caribbean, Northamerica descent |
|  |  | 4_68_BRCA1 | 43 | present | Asian, European, African, LATAM/caribbean, Northamerica descent |
|  |  | 4_69_BRCA1 | 60 | present | Asian, European, African, LATAM/caribbean, Northamerica descent |
|  |  | 4_70_BRCA1 | 44 | present | Asian, European, African, LATAM/caribbean, Northamerica descent |
|  |  | 4_71_BRCA1 | 61 | present | Asian, European, African, LATAM/caribbean, Northamerica descent |
|  |  | 4_72_BRCA1 | 71 | present | Asian, European, African, LATAM/caribbean, Northamerica descent |
|  |  | 4_73_BRCA1 | 48 | present | Asian, European, African, LATAM/caribbean, Northamerica descent |
|  |  | 4_74_BRCA1 | 44 | present | Asian, European, African, LATAM/caribbean, Northamerica descent |
|  |  | 4_75_BRCA1 | 59 | present | Asian, European, African, LATAM/caribbean, Northamerica descent |
|  |  | 4_76_BRCA1 | 44 | present | Asian, European, African, LATAM/caribbean, Northamerica descent |
| Felicio et al., 2021 (54) | Brazil | 5_1_CHEK2 | 21 | present | Multiple ethnie |
| Adaniel et al., 2019 (56) | Chile | 6_1_RAD51D | 50 | present | Multiple ethnie |
| Moreira et al., 2019 (55) | Brazil | 7_1_BRCA2 | 64 | NR | European, LATAM/caribbean,Northamerica |
|  |  | 7_2_BRCA1 | 47 | breast | European, LATAM/caribbean,Northamerica |
|  |  | 7_3_CHEK2 | 44 | NR | European, LATAM/caribbean,Northamerica |
| Cock-Rada et al., 2017 (58) | Colombia | 8_1_BRCA1 | 47 | ovarian | LATAM/caribbean |

**Supplemental Table 6. Individual-level data of ovarian cancer patients carrying HRR variants across included studies.** Age_dx, age at diagnosis; Familial_history_cancer, reported family history of cancer; ancestry_major, major ancestry or ethnicity; NR, not reported; LATAM, Latin American; HRR, homologous recombination repair.

| **study_id (ref)** | | **Country** | **ID_case** | **Gene** | **Transcript_curation** | **HGVS coding** | **HGVS Protein** | **Variant origin (germline/somatic)** | **Type** | **ACMG_Class** | **Curation** |  |
| --- | --- | --- | --- | --- | --- | --- | --- | --- | --- | --- | --- | --- |
| Pérez-Ibave et al., 2023 (51) | | Mexico | 1_1_BRCA1 | *BRCA1* | NM_007294.4 | c.211A>G | p.Arg71Gly | Germline | Missense | Pathogenic | Pass |  |
|  |  | Mexico | 1_2_BRCA1 | *BRCA1* | NM_007294.4 | c.115T>A | p.Cys39Ser | Germline | Missense | Pathogenic | Pass |  |
| De Carvalho et al., 2023 (52) | | Brazil | 2_1_BRCA1 | *BRCA1* | NM_007294.4 | c.470_471del | p.Ser157Ter | Germline | Nonsense | Pathogenic | Pass |  |
| De Oliveira et al., 2022 (57) | | Brazil | 3_1_BRCA2 | *BRCA2* | NM_000059.4 | c.7645dup | p.Cys2549LeufsTer5 | Germline | Frameshift | Likely pathogenic | Pass |  |
|  |  |  | 3_2_BRCA2 | *BRCA2* | NM_000059.4 | c.4648G>T | p.Glu1550Ter | Germline | Nonsense | Pathogenic | Pass |  |
|  |  |  | 3_3_BRCA1 | *BRCA1* | NM_007294.4 | c.3331_3334del | p.Gln1111AsnfsTer5 | Germline | Frameshift | Pathogenic | Pass |  |
|  |  |  | 3_4_BRCA1 | *BRCA1* | NM_007294.4 | c.3331_3334del | p.Gln1111AsnfsTer5 | Germline | Frameshift | Pathogenic | Pass |  |
|  |  |  | 3_5_BRCA1 | *BRCA1* | NM_007294.4 | c.3331_3334del | p.Gln1111AsnfsTer5 | Germline | Frameshift | Pathogenic | Pass |  |
|  |  |  | 3_6_BRCA1 | *BRCA1* | NM_007294.4 | c.5074+2T>C | p.? | Germline | Splice site (donor) | Pathogenic | Pass |  |
|  |  |  | 3_7_BRCA1 | *BRCA1* | NM_007294.4 | c.4484G>A | p.Arg1495Lys | Germline | Missense | Pathogenic | Pass |  |
|  |  |  | 3_8_BRCA1 | *BRCA1* | NM_007294.4 | c.5266dup | p.Gln1756ProfsTer74 | Germline | Frameshift | Pathogenic | Pass |  |
|  |  |  | 3_9_BRCA1 | *BRCA1* | NM_007294.4 | c.1687C>T | p.Gln563Ter | Germline | Nonsense | Pathogenic | Pass |  |
|  |  |  | 3_10_CHEK2 | *CHEK2* | NM_007194.4 | c.470T>C | p.Ile157Thr | Germline | Missense | Likely pathogenic | Pass |  |
|  |  |  | 3_11_BARD1 | *BARD1* | NM_007194.4 | c.176_177del | p.Glu59AlafsTer8 | Germline | Frameshift | Pathogenic | Pass |  |
| Solano et al., 2021 (53) | | Argentina | 4_1_BRCA2 | *BRCA2* | NM_000059.4 | c.517G>T | p.Gly173Cys | Germline | Missense | Likely pathogenic | Pass |  |
|  |  |  | 4_2_BRCA2 | *BRCA2* | NM_000059.4 | c.1337T>A | p.Leu446Ter | Germline | Nonsense | Pathogenic | Pass |  |
|  |  |  | 4_3_BRCA2 | *BRCA2* | NM_000059.4 | c.1597del | p.Thr533LeufsTer25 | Germline | Frameshift | Pathogenic | Pass |  |
|  |  |  | 4_4_BRCA2 | *BRCA2* | NM_000059.4 | c.1597del | p.Thr533LeufsTer25 | Germline | Frameshift | Pathogenic | Pass |  |
|  |  |  | 4_5_BRCA2 | *BRCA2* | NM_000059.4 | c.1909+1G>A | p.? | Germline | Splice site (donor) | Pathogenic | Pass |  |
|  |  |  | 4_6_BRCA2 | *BRCA2* | NM_000059.4 | c.1909+1G>A | p.? | Germline | Splice site (donor) | Pathogenic | Pass |  |
|  |  |  | 4_7_BRCA2 | *BRCA2* | NM_000059.4 | c.1909+1G>A | p.? | Germline | Splice site (donor) | Pathogenic | Pass |  |
|  |  |  | 4_8_BRCA2 | *BRCA2* | NM_000059.4 | c.2094del | p.Gln699SerfsTer31 | Germline | Frameshift | Pathogenic | Pass |  |
|  |  |  | 4_9_BRCA2 | *BRCA2* | NM_000059.4 | c.2133C>A | p.Cys711Ter | Germline | Nonsense | Pathogenic | Pass |  |
|  |  |  | 4_10_BRCA2 | *BRCA2* | NM_000059.4 | c.2657delA | p.Asn886MetfsTer9 | Germline | Frameshift | Pathogenic | Pass |  |
|  |  |  | 4_11_BRCA2 | *BRCA2* | NM_000059.4 | c.2808_2811del | p.Ala938ProfsTer21 | Germline | Frameshift | Pathogenic | Pass |  |
|  |  |  | 4_12_BRCA2 | *BRCA2* | NM_000059.4 | c.2860G>T | p.Glu954Ter | Germline | Nonsense | Pathogenic | Pass |  |
|  |  |  | 4_13_BRCA2 | *BRCA2* | NM_000059.4 | c.3545_3546del | p.Phe1182Ter | Germline | Nonsense | Pathogenic | Pass |  |
|  |  |  | 4_14_BRCA2 | *BRCA2* | NM_000059.4 | c.4222C>T | p.Gln1408Ter | Germline | Nonsense | Pathogenic | Pass |  |
|  |  |  | 4_15_BRCA2 | *BRCA2* | NM_000059.4 | c.4284dup | p.Gln1429SerfsTer9 | Germline | Frameshift | Pathogenic | Pass |  |
|  |  |  | 4_16_BRCA2 | *BRCA2* | NM_000059.4 | c.4419del | p.Asn1473LysfsTer6 | Germline | Frameshift | Pathogenic | Pass |  |
|  |  |  | 4_17_BRCA2 | *BRCA2* | NM_000059.4 | c.4964dup | p.Tyr1655Ter | Germline | Nonsense | Pathogenic | Pass |  |
|  |  |  | 4_18_BRCA2 | *BRCA2* | NM_000059.4 | c.5073dup | p.Trp1692MetfsTer3 | Germline | Frameshift | Pathogenic | Pass |  |
|  |  |  | 4_19_BRCA2 | *BRCA2* | NM_000059.4 | c.5130_5133del | p.Tyr1710_Val1711delinsTer | Germline | Nonsense | Pathogenic | Pass |  |
|  |  |  | 4_20_BRCA2 | *BRCA2* | NM_000059.4 | c.5146_5149del | p.Tyr1716LysfsTer8 | Germline | Frameshift | Pathogenic | Pass |  |
|  |  |  | 4_21_BRCA2 | *BRCA2* | NM_000059.4 | c.5146_5149del | p.Tyr1716LysfsTer8 | Germline | Frameshift | Pathogenic | Pass |  |
|  |  |  | 4_22_BRCA2 | *BRCA2* | NM_000059.4 | c.5351dup | p.Asn1784LysfsTer3 | Germline | Frameshift | Pathogenic | Pass |  |
|  |  |  | 4_23_BRCA2 | *BRCA2* | NM_000059.4 | c.5351dup | p.Asn1784LysfsTer3 | Germline | Frameshift | Pathogenic | Pass |  |
|  |  |  | 4_24_BRCA2 | *BRCA2* | NM_000059.4 | c.5351dup | p.Asn1784LysfsTer3 | Germline | Frameshift | Pathogenic | Pass |  |
|  |  |  | 4_25_BRCA2 | *BRCA2* | NM_000059.4 | c.5351dup | p.Asn1784LysfsTer3 | Germline | Frameshift | Pathogenic | Pass |  |
|  |  |  | 4_26_BRCA2 | *BRCA2* | NM_000059.4 | c.5723_5724del | p.Leu1908ArgfsTer2 | Germline | Frameshift | Pathogenic | Pass |  |
|  |  |  | 4_27_BRCA2 | *BRCA2* | NM_000059.4 | c.5959C>T | p.Gln1987Ter | Germline | Nonsense | Pathogenic | Pass |  |
|  |  |  | 4_28_BRCA2 | *BRCA2* | NM_000059.4 | c.6275_6276del | p.Leu2092ProfsTer7 | Germline | Frameshift | Pathogenic | Pass |  |
|  |  |  | 4_29_BRCA2 | *BRCA2* | NM_000059.4 | c.6395T>G | p.Leu2132Ter | Germline | Nonsense | Pathogenic | Pass |  |
|  |  |  | 4_30_BRCA2 | *BRCA2* | NM_000059.4 | c.6486_6489del | p.Lys2162AsnfsTer5 | Germline | Frameshift | Pathogenic | Pass |  |
|  |  |  | 4_31_BRCA2 | *BRCA2* | NM_000059.4 | c.6596del | p.Thr2199IlefsTer7 | Germline | Frameshift | Pathogenic | Pass |  |
|  |  |  | 4_32_BRCA2 | *BRCA2* | NM_000059.4 | c.7976G>A | p.Arg2659Lys | Germline | Missense | Likely pathogenic | Pass |  |
|  |  |  | 4_33_BRCA2 | *BRCA2* | NM_000059.4 | c.8488-1G>T | p.? | Germline | Splice site (acceptor) | Pathogenic | Pass |  |
|  |  |  | 4_34_BRCA2 | *BRCA2* | NM_000059.4 | c.8488-1G>T | p.? | Germline | Splice site (acceptor) | Pathogenic | Pass |  |
|  |  |  | 4_35_BRCA2 | *BRCA2* | NM_000059.4 | c.8987T>A | p.Leu2996Ter | Germline | Nonsense | Pathogenic | Pass |  |
|  |  |  | 4_36_BRCA2 | *BRCA2* | NM_000059.4 | c.1909+1G>A | p.? | Germline | Splice site (donor) | Pathogenic | Pass |  |
|  |  |  | 4_37_BRCA1 | *BRCA1* | NM_007294.4 | c.211A>G | p.Arg71Gly | Germline | Missense | Pathogenic | Pass |  |
|  |  |  | 4_38_BRCA1 | *BRCA1* | NM_007294.4 | c.211A>G | p.Arg71Gly | Germline | Missense | Pathogenic | Pass |  |
|  |  |  | 4_39_BRCA1 | *BRCA1* | NM_007294.4 | c.211A>G | p.Arg71Gly | Germline | Missense | Pathogenic | Pass |  |
|  |  |  | 4_40_BRCA1 | *BRCA1* | NM_007294.4 | c.68_69del | p.Glu23ValfsTer17 | Germline | Frameshift | Pathogenic | Pass |  |
|  |  |  | 4_41_BRCA1 | *BRCA1* | NM_007294.4 | c.134+1G>C | p.? | Germline | Splice site (donor) | Pathogenic | Pass |  |
|  |  |  | 4_42_BRCA1 | *BRCA1* | NM_007294.4 | c.181T>G | p. Cys61Gly | Germline | Missense | Pathogenic | Pass |  |
|  |  |  | 4_43_BRCA1 | *BRCA1* | NM_007294.4 | c.190T>C | p.Cys64Arg | Germline | Missense | Pathogenic | Pass |  |
|  |  |  | 4_44_BRCA1 | *BRCA1* | NM_007294.4 | c.250G>T | p.Glu84Ter | Germline | Nonsense | Pathogenic | Pass |  |
|  |  |  | 4_45_BRCA1 | *BRCA1* | NM_007294.4 | c.547+2T>A | p.? | Germline | Splice site (donor) | Pathogenic | Pass |  |
|  |  |  | 4_46_BRCA1 | *BRCA1* | NM_007294.4 | c.835del | p.His279MetfsTer19 | Germline | Frameshift | Pathogenic | Pass |  |
|  |  |  | 4_47_BRCA1 | *BRCA1* | NM_007294.4 | c.876_879del | p.Thr293LysfsTer4 | Germline | Frameshift | Pathogenic | Pass |  |
|  |  |  | 4_48_BRCA1 | *BRCA1* | NM_007294.4 | c.1360_1361del | p.Ser454Ter | Germline | Nonsense | Pathogenic | Pass |  |
|  |  |  | 4_49_BRCA1 | *BRCA1* | NM_007294.4 | c.1504_1507del | p.Leu502SerfsTer29 | Germline | Frameshift | Pathogenic | Pass |  |
|  |  |  | 4_50_BRCA1 | *BRCA1* | NM_007294.4 | c.1729_1730del | p.Glu577IlefsTer8 | Germline | Frameshift | Pathogenic | Pass |  |
|  |  |  | 4_51_BRCA1 | *BRCA1* | NM_007294.4 | c.1817del | p.Pro606LeufsTer6 | Germline | Frameshift | Pathogenic | Pass |  |
|  |  |  | 4_52_BRCA1 | *BRCA1* | NM_007294.4 | c.1953dup | p.Lys652GlufsTer21 | Germline | Frameshift | Pathogenic | Pass |  |
|  |  |  | 4_53_BRCA1 | *BRCA1* | NM_007294.4 | c.2005dup | p.Met669AsnfsTer4 | Germline | Frameshift | Likely pathogenic | Pass |  |
|  |  |  | 4_54_BRCA1 | *BRCA1* | NM_007294.4 | c.2411_2412del | p.Gln804LeufsTer5 | Germline | Frameshift | Pathogenic | Pass |  |
|  |  |  | 4_55_BRCA1 | *BRCA1* | NM_007294.4 | c.2486_2487del | p.Phe829Ter | Germline | Nonsense | Pathogenic | Pass |  |
|  |  |  | 4_56_BRCA1 | *BRCA1* | NM_007294.4 | c.2487del | p.Phe829LeufsTer17 | Germline | Frameshift | Pathogenic | Pass |  |
|  |  |  | 4_57_BRCA1 | *BRCA1* | NM_007294.4 | c.2487del | p.Phe829LeufsTer17 | Germline | Frameshift | Pathogenic | Pass |  |
|  |  |  | 4_58_BRCA1 | *BRCA1* | NM_007294.4 | c.2568T>G | p.Tyr856Ter | Germline | Nonsense | Pathogenic | Pass |  |
|  |  |  | 4_59_BRCA1 | *BRCA1* | NM_007294.4 | c.3309dup | p.Lys1104Ter | Germline | Nonsense | Likely pathogenic | Pass |  |
|  |  |  | 4_60_BRCA1 | *BRCA1* | NM_007294.4 | c.3756_3759del | p.Ser1253ArgfsTer10 | Germline | Frameshift | Pathogenic | Pass |  |
|  |  |  | 4_61_BRCA1 | *BRCA1* | NM_007294.4 | c.3756_3759del | p.Ser1253ArgfsTer10 | Germline | Frameshift | Pathogenic | Pass |  |
|  |  |  | 4_62_BRCA1 | *BRCA1* | NM_007294.4 | c.3758_3759del | p.Ser1253Ter | Germline | Nonsense | Pathogenic | Pass |  |
|  |  |  | 4_63_BRCA1 | *BRCA1* | NM_007294.4 | c.4042G>T | p.Gly1348Ter | Germline | Nonsense | Pathogenic | Pass |  |
|  |  |  | 4_64_BRCA1 | *BRCA1* | NM_007294.4 | c.4117G>T | p.Glu1373Ter | Germline | Nonsense | Pathogenic | Pass |  |
|  |  |  | 4_65_BRCA1 | *BRCA1* | NM_007294.4 | c.4484G>A | p.Arg1495Lys | Germline | Missense | Pathogenic | Pass |  |
|  |  |  | 4_66_BRCA1 | *BRCA1* | NM_007294.4 | c.4688dup | p.Tyr1563Ter | Germline | Nonsense | Pathogenic | Pass |  |
|  |  |  | 4_67_BRCA1 | *BRCA1* | NM_007294.4 | c.4964_4982del | p.Ser1655TyrfsTer16 | Germline | Frameshift | Pathogenic | Pass |  |
|  |  |  | 4_68_BRCA1 | *BRCA1* | NM_007294.4 | c.4987-1G>A | p.? | Germline | Splice site (acceptor) | Pathogenic | Pass |  |
|  |  |  | 4_69_BRCA1 | *BRCA1* | NM_007294.4 | c.5030_5033del | p.Thr1677IlefsTer2 | Germline | Frameshift | Pathogenic | Pass |  |
|  |  |  | 4_70_BRCA1 | *BRCA1* | NM_007294.4 | c.5095C>T | p.Arg1699Trp | Germline | Missense | Pathogenic | Pass |  |
|  |  |  | 4_71_BRCA1 | *BRCA1* | NM_007294.4 | c.5266dup | p.Gln1756ProfsTer74 | Germline | Frameshift | Pathogenic | Pass |  |
|  |  |  | 4_72_BRCA1 | *BRCA1* | NM_007294.4 | c.5266dup | p.Gln1756ProfsTer74 | Germline | Frameshift | Pathogenic | Pass |  |
|  |  |  | 4_73_BRCA1 | *BRCA1* | NM_007294.4 | c.5266dup | p.Gln1756ProfsTer74 | Germline | Frameshift | Pathogenic | Pass |  |
|  |  |  | 4_74_BRCA1 | *BRCA1* | NM_007294.4 | c.5434C>G | p.Pro1812Ala | Germline | Missense | Pathogenic | Pass |  |
|  |  |  | 4_75_BRCA1 | *BRCA1* | NM_007294.4 | c.5444G>A | p.Trp1815Ter | Germline | Nonsense | Pathogenic | Pass |  |
|  |  |  | 4_76_BRCA1 | *BRCA1* | NM_007294.4 | c.5509T>A | p.Trp1837Arg | Germline | Missense | Pathogenic | Pass |  |
|  |  |  | 4_77_BRCA1 | *BRCA1* | NM_007294.4 | c.(?_-232)_(*1383_?)del | p.? | Germline | INDEL | Pathogenic | Pass |  |
| Felicio et al., 2021 (54) | | Brazil | 5_1_CHEK2 | *CHEK2* | NM_007194.4 | c.538C>T | p. Arg180Cys | Germline | Missense | Likely pathogenic | Not pass* |  |
| Adaniel et al., 2019 (56) | | Chile | 6_1_RAD51D | *RAD51D* | NM_002878.4 | c.216C>A | p.Tyr72Ter | Germline | Nonsense | Likely pathogenic | Pass |  |
| Moreira et al., 2019 (55) | | Brazil | 7_1_BRCA2 | *BRCA2* | NM_000059.4 | c.4829_4830del | p.Val1610GlyfsTer4 | Germline | Frameshift | Pathogenic | Pass |  |
|  |  |  | 7_2_BRCA1 | *BRCA1* | NM_007294.4 | c.3331_3334del | p.Gln1111AsnfsTer5 | Germline | Frameshift | Pathogenic | Pass |  |
|  |  |  | 7_3_CHEK2 | *CHEK2* | NM_007194.4 | c.485A>G | p.Asp162Gly | Germline | Missense | Likely pathogenic | Pass |  |
| Cock-Rada et al., 2017 (58) | | Colombia | 8_1_BRCA1 | *BRCA1* | NM_007294.4 | c.1674del | p.Gly559ValfsTer13 | Germline | Frameshift | Pathogenic | Pass |  |
|  | **Supplemental Table 7. Curated germline HRR gene variants reported in Latin American ovarian cancer studies.** *This variant was curated, leading to a reclassification based on the application of the following criteria: PM5_supporting (an alternative variant classified as likely pathogenic was identified at the same residue), and PP3 (in silico predictors support a deleterious effect). Consequently, the variant was reclassified as a variant of uncertain clinical significance (VUS). | | | | | | | | | | | |

| A. Heterogeneity and sensitivity analyses | | | | | |
| --- | --- | --- | --- | --- | --- |
| Analysis | **k** | **Pooled prevalence % (95% CI)** | **I² % (95% CI)** | **τ²** | **p (Q)** |
| Primary random-effects model | 7 | 17 (9–26) | 56.2 (0.0–81.2) | 0 | 0.033 |
| Excluding micro-cohorts (n < 5) | 5 | 20 (16–23) | 0 (0.0–79.2) | 0 | 0.837 |
| Random-intercept logistic model | 7 | 22.2 (17.7–27.4) | σ² = 0 | 0 | – |
| B. Exploratory subgroup analyses | | | | | |
| Moderator | **k** | **Subgroup** | **Carriers / n** | **Prevalence % (95% CI)** | **p** |
| Recruitment setting | 2 | Hereditary cancer clinic | 3 / 19 | 15.8 (3.4–39.6) | 0.48 |
|  | 5 | Hospital-based oncology | 93 / 414 | 22.5 (18.5–26.8) |  |
| Country | 3 | Brazil | 15 / 77 | 19.5 (11.3–30.1) | 0.53 |
|  | 4 | Other Latin American | 81 / 356 | 22.8 (18.5–27.5) |  |
| Panel breadth | 3 | Comprehensive (WES or ≥30 genes) | 90 / 413 | 21.8 (17.9–26.1) | 0.40 |
|  | 4 | Limited or targeted | 6 / 20 | 30.0 (11.9–54.3) |  |
| Cohort size | 5 | n < 20 | 8 / 29 | 27.6 (12.7–47.2) | 0.48 |
|  | 2 | n ≥ 20 | 88 / 404 | 21.8 (17.9–26.1) |  |
| Sequencing platform | 4 | Illumina | 91 / 419 | 21.7 (17.9–26.0) | Not assessable |
|  | 2 | Not reported | 2 / 11 | 18.2 (2.3–51.8) |  |
|  | 1 | Sanger | 3 / 3 | 100 (29.2–100) |  |

**Supplemental Table 8. Exploration of between-study heterogeneity in the pooled prevalence of P/LP germline HRR variants.** (A) reports heterogeneity for the primary model, for the sensitivity analysis excluding the two cohorts of fewer than five patients, and for a random-intercept logistic model fitted to accommodate sparse data and boundary proportions. The confidence interval for I² includes zero, reflecting the imprecision inherent to a synthesis of seven cohorts. (B) reports subgroup analyses; because several subgroups contained cohorts of one to three patients, for which back-transformed pooled estimates are unstable, subgroups are summarised as crude prevalences with exact binomial (Clopper-Pearson) confidence intervals and compared by likelihood-ratio test. Sequencing platform could not be assessed because it was not reported in two cohorts and only one cohort used a non-Illumina platform. With one to five studies per subgroup these comparisons have very limited statistical power and are exploratory. k, number of cohorts; CI, confidence interval; WES, whole-exome sequencing.

**Supplemental Figure 1. Risk of bias assessment across included studies using the Joanna Briggs Institute (JBI) critical appraisal tool.**

**Supplemental Figure 2. Risk of bias assessment of individual studies using the Joanna Briggs Institute (JBI) critical appraisal tool.**
